# Coordinated protein and DNA conformational changes govern mismatch repair initiation by MutS

**DOI:** 10.1093/nar/gky865

**Published:** 2018-10-01

**Authors:** Sharonda J LeBlanc, Jacob W Gauer, Pengyu Hao, Brandon C Case, Manju M Hingorani, Keith R Weninger, Dorothy A Erie

**Affiliations:** 1 Department of Chemistry, University of North Carolina at Chapel Hill, Chapel Hill, NC 27599, USA; 2 Department of Physics, North Carolina State University, Raleigh, NC 27695, USA; 3 Molecular Biology and Biochemistry Department, Wesleyan University, Middletown, CT 06459, USA; 4 Lineberger Comprehensive Cancer Center, University of North Carolina at Chapel Hill, Chapel Hill, NC 27599, USA

## Abstract

MutS homologs identify base-pairing errors made in DNA during replication and initiate their repair. In the presence of adenosine triphosphate, MutS induces DNA bending upon mismatch recognition and subsequently undergoes conformational transitions that promote its interaction with MutL to signal repair. In the absence of MutL, these transitions lead to formation of a MutS mobile clamp that can move along the DNA. Previous single-molecule FRET (smFRET) studies characterized the dynamics of MutS DNA-binding domains during these transitions. Here, we use protein–DNA and DNA–DNA smFRET to monitor DNA conformational changes, and we use kinetic analyses to correlate DNA and protein conformational changes to one another and to the steps on the pathway to mobile clamp formation. The results reveal multiple sequential structural changes in both MutS and DNA, and they suggest that DNA dynamics play a critical role in the formation of the MutS mobile clamp. Taking these findings together with data from our previous studies, we propose a unified model of coordinated MutS and DNA conformational changes wherein initiation of mismatch repair is governed by a balance of DNA bending/unbending energetics and MutS conformational changes coupled to its nucleotide binding properties.

## INTRODUCTION

Maintaining integrity of genetic information is essential during DNA replication. DNA synthesis is inherently accurate due to the high fidelity of DNA polymerases, which have an error frequency of ∼10^−7^. On rare occasions, bases are misincorporated, resulting in non-Watson/Crick base pairs or insertion/deletion loop (IDL) errors (1–3). DNA mismatch repair (MMR) increases the fidelity of DNA replication up to 1000-fold by correcting base-base mismatches and IDLs introduced during DNA replication (1,2,4,5). Uncorrected errors can lead to mutations, genomic instability and cancer (1,6,7). In humans, mutations in MMR genes are responsible for Lynch Syndrome and linked to >80% of hereditary non-polyposis colorectal cancer cases as well as many sporadic cancers (7–10).

MMR is highly conserved across all organisms and has been reconstituted and investigated *in vitro*, mainly with *Escherichia coli*, yeast and human proteins (3,11–13). In all organisms, MMR is initiated by binding of MutS homologs to a base–base mismatch or IDL followed by adenosine triphosphate (ATP)-dependent conformational changes to form a mobile clamp that can move along the DNA (14). Although the function of mobile clamp state remains unclear, ATP activation of MutS homologs is required for the downstream events that lead to repair. Specifically, the ATP-activated MutS homolog recruits MutL homologs and promotes removal of the error-containing DNA segment and resynthesis (1–3). MutS and MutL homologs are heterodimers (MutSα [MSH2:MSH6]; MutLα [MLH1:PMS2]) in eukaryotes and homodimers in prokaryotes; however, the prokaryotic MutS adopts asymmetric properties upon nucleotide and/or mismatch binding. Specifically, only one subunit interacts with the mismatch, and nucleotide binding to one subunit alters the affinity of nucleotides for the other subunit (15,16). Due to its similarity to eukaryotic MMR, the *Thermus aquaticus* (*Taq*) MMR system is a good model system to aid our understanding of human MMR (17–26).

**Figure 1.**
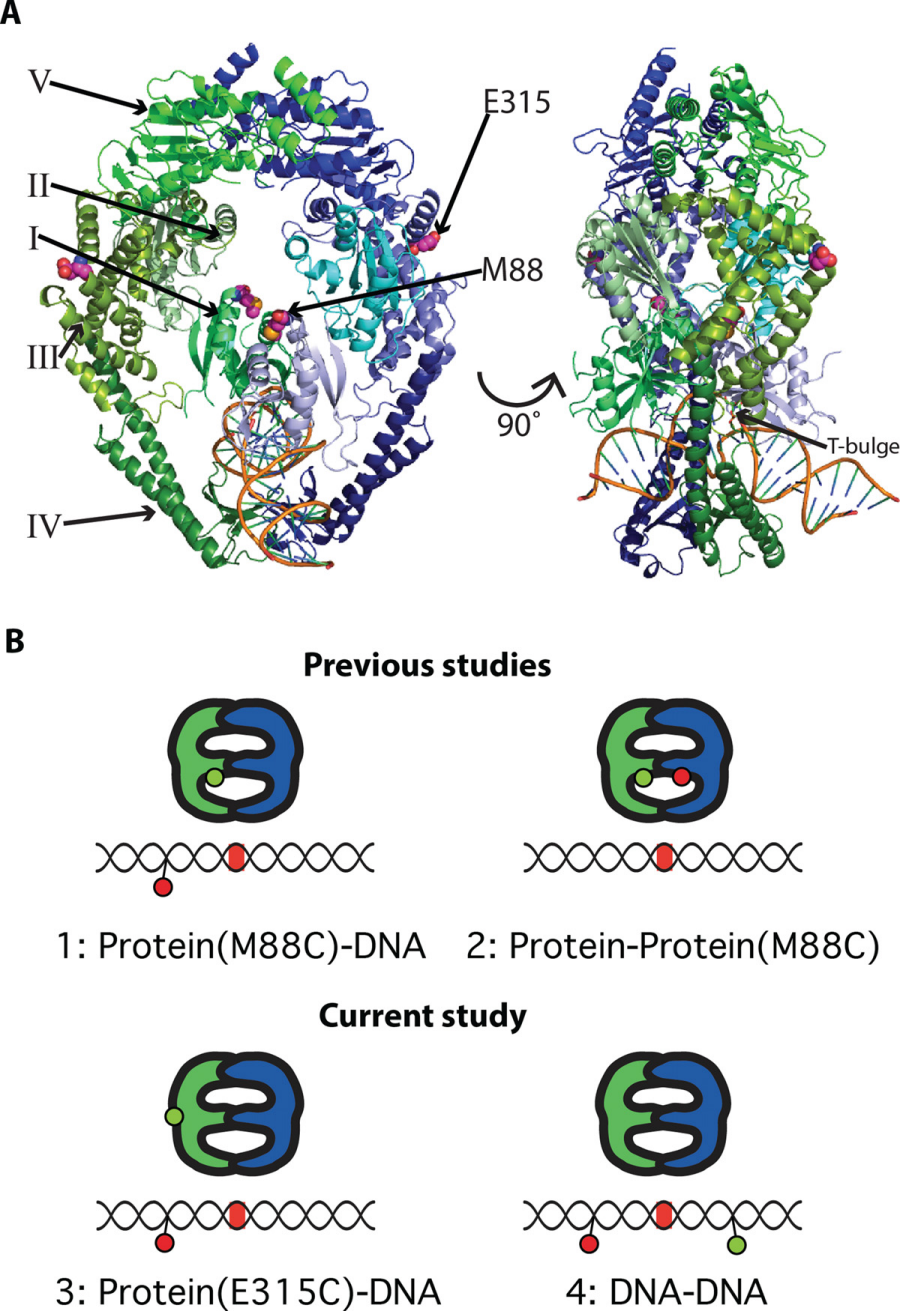
Labeling strategies for smFRET experiments. (**A**) Crystal structure of *Taq* MutS homodimer (PDB ID 1EWQ). The monomers are colored blue (subunit A) and green (subunit B). Domains are represented by different shades of blue or green. The DNA backbone is gold. Domains I through V and dye label sites (M88 and E315) are indicated. (**B**) Cartoons show labeling strategies used to study conformational dynamics of *Taq* MutS–DNA interactions. MutS is depicted with blue and green monomer units. Green and red circles represent donor and acceptor fluorophores, respectively. The DNA segments contain a mismatch (red line). The label below each cartoon indicates the donor–acceptor positions. 1: One Domain I of *Taq* MutS labeled at M88C (a dynamic position on MutS) with AF555 (donor) and a 550-bp T-bulge substrate labeled with Cy5 (acceptor) 9 bp 3′ from the mismatch, used to monitor interaction with the mismatch DNA in previous studies (23,26). 2: One donor and one acceptor (AF555 and AF647) on M88C of Domains I used to monitor the dynamics of the DNA binding domains in previous studies (23,26). 3: One Domain I of *Taq* MutS labeled at E315C (a relatively non-mobile site on MutS) with AF555 (donor) and a 550-bp T-bulge substrate labeled with Cy5 (acceptor) 9 bp 3′ from the mismatch, used to monitor interaction with the mismatch DNA in the current study. 4: 68-bp T-bulge DNA substrate labeled with a TAMRA and Cy5 FRET pair separated by 19 bp, used in this study to directly probe the DNA conformation upon initial mismatch recognition and mobile clamp formation.

The crystal structures of *Taq* and *E. coli* MutS and human MutSα bound to heteroduplex DNAs containing several different mismatches have shed light onto the interactions that govern mismatch recognition (17,18,27–29). MutS is an asymmetric disc-shaped dimer containing two channels separated by Domains I, with the DNA in the lower channel and the ATPase sites at the top of the upper channel (Domains V), ∼60Å from the DNA (Figure 1A). The DNA binding domains (Domains I and IV) from the two subunits encircle the DNA and make mostly non-specific contacts (Figure 1A). The only specific contacts made between MutS and the mismatch are by Phe and Glu from one subunit, which are in the Phe-Xaa-Glu motif that is conserved in prokaryotes (17,27) and in the eukaryotic MutS homolog MSH6 (29). For mismatch recognition, the conserved Phe stacks with a mispaired or inserted base, which is rotated slightly into the minor groove, and the Glu forms a hydrogen bond with the base (17,27,29). All of the MutS–mismatch–DNA structures show DNA kinked with a 45°-60° bend angle, suggesting that DNA bending coupled with local flexibility at the mismatch may be important in mismatch recognition (17,27,30,31). Atomic force microscopy (AFM) and Förster/fluorescence resonance energy transfer (FRET) (single molecule and bulk) studies have identified multiple DNA conformations in MutS–mismatch DNA complexes ranging from significantly bent to unbent (or slightly bent) (19–21,24,32–34). Furthermore, previous studies revealed that the ability of a MutS–DNA complex to transition from the bent to unbent state correlates with its propensity to signal repair (20,24). Although these findings suggest that MutS-induced DNA bending and unbending partially controls MMR, the studies were conducted in the absence of ATP, which is required for both initiation and progression of MMR (1,15,16,18,23,35–41). ATP is known to induce conformational changes in the MutS–DNA complex that promote the transition from mismatch bound state to states that signal repair. In addition, ATP-induced DNA unbending by MutS has been observed (32,42). Nevertheless, our knowledge of the ATP-dependent pathway to form the signaling state is incomplete. Here, we address this gap by characterizing MutS-induced DNA bending in the presence of ATP by following the dynamic DNA conformational changes as MutS transitions from a mismatch recognition complex to a mobile clamp state.

We use single-molecule FRET (smFRET) to monitor the dynamics of *Taq* MutS-induced DNA bending/unbending during mismatch recognition and mobile clamp formation. This study complements our previous smFRET-based investigation of dynamic conformational changes in *Taq* MutS during the same process (23,26). FRET between donor-labeled MutS and acceptor-labeled mismatch DNA reports on the position of a MutS domain relative to the mismatch, intra-protein FRET between donor and acceptor dyes on mobile Domains I reports protein conformational changes, and FRET between donor and acceptor dyes flanking the mismatch directly reports dynamic DNA bending/unbending (Figure 1B). Correlating the data from these experiments allows us to characterize the protein and DNA conformational changes along the observed sequential steps in the pathway leading to mobile clamp formation. As seen with our previous studies on protein conformational changes, the current experiments monitoring DNA conformations indicate a preferred pathway of DNA bending states. Together, these results offer a unified model of the protein and DNA conformational changes that govern initiation of MMR and reveal a critical role for DNA dynamics in the process.

## MATERIALS AND METHODS

### Proteins

The C42A/E315C *Taq* MutS variant plasmid was prepared by QuikChange site-directed mutagenesis (Agilent). All MutS proteins were expressed in *E. coli* BL21(DE3) cells, and purified by (NH_4_)_2_SO_4_ precipitation (24%) and dialysis against buffer A (50 mM Tris–HCl, pH 7.5, 50 mM NaCl, 1 mM ethylenediaminetetraacetic acid, 5% w/v glycerol), followed by Q-sepharose chromatography (50–350 mM NaCl gradient) in buffer A (23,34,43). C42A/E315C MutS was labeled with Alexa Fluor^®^ 555 (AF555) fluorophore with 90% labeling efficiency on a monomer basis, as described previously (23,34). Section I of the Supplementary Data and Supplementary Table S1 describe how the labeling efficiency was calculated.

### DNA substrates

The 68-base pair (bp) substrate utilized for DNA bending experiments was prepared with the following fluorescently labeled DNAs (IDT): b-CTC TAG AGG ATC CGC TGA GGC CAG CTG AGG CCT GGC TGA GGA TTG CTG A(T*)G AAT TCA CTG GCC GTC G; dig-CGA CGG CCA GTG AAT TCA TCA GCA ATC **T**CT CAG CCA G/iCy5/GC CTC AGC TGG CCT CAG CGG ATC CTC TAG AG. The ‘b’ represents 5′ biotinylation and ‘dig’ represents 5′ digoxigenin modification to facilitate blocking of the free end (DNA was *not* end-blocked in this study). The bold and underlined T is a single thymine insertion (T-bulge), T* is linked to a TAMRA dye (IDT provided TAMRA NHS Ester through a dT base) and /iCy5/ marks an internal Cy5 label (synthesized by IDT). A graphical representation of the duplex DNA with attached dyes is shown in Supplementary Figure S1. The sequence was based on DNA substrates used in previous studies, with the only change being substitution of T* to allow for labeling (23,26). The fluorophore separation (19 bp) was based on previous studies (21,24). The duplex substrate was prepared by heating the complementary strands to 94°C for 2 min and then slowly cooling to room temperature. Annealed substrates were stored at 4°C for up to 2 weeks. The 550-bp DNA substrate utilized for AF555-MutS-E315C:Cy5-DNA experiments was prepared as described previously (26).

### Dye mobility simulation

We used Crystallography & NMR System (CNS) software to simulate the position of dyes attached at E315C on MutS (PDB code: 1EWQ; Supplementary Figure S5). Cy3 and Cy5 dyes were used in the simulation due to the lack of a published structure for AF555 (44–46). The simulation yielded average positions of the dye atoms, and the mass centers of the dye molecules were marked for distance measurements. Conjugation of dye to DNA was accomplished based on the internal dye structure published by IDT (47).

### Surface preparation for experiments

Quartz slides and glass coverslips were cleaned by bath sonication in acetone, ethanol and KOH in that order, then rinsed and stored in water. For protein–DNA FRET experiments, functionalized PEG-passivated surfaces were prepared as follows. Methoxy-poly (ethylene glycol)–silane, MW 2000 (Laysan Bio) and biotin-poly (ethylene glycol)-silane, MW 3400 (Laysan Bio) were aliquoted in 20 and 2 mg quantities in a glove box. Both slides and coverslips were dried with compressed air. A total of 80 μl of water was added to the methoxy-PEG-silane aliquot, vortexed to mix and air bubbles were removed by short centrifugation. A 2 mg aliquot of biotin-PEG-silane was dissolved with 10 μl of water. A total of 1 μl of the biotin-PEG-silane was added to the methoxy-PEG-silane solution to obtain a 1:100 biotin/non-biotin ratio. The PEG-silane mixture (∼40 μl) was applied to the quartz slide and covered with the coverslip, avoiding introduction of air bubbles. The slide was incubated overnight inside a small box containing water to saturate humidity and prevent evaporation of the PEG solution. The coverslip was removed and both slide and coverslip rinsed thoroughly with deionized water on the functionalized surface and allowed to dry in air. The slides were treated with a second round of methoxy-PEG-silane to improve blocking (48). In both types of experiments, flow channels were constructed from double sided tape between the slide and coverslip. For DNA–DNA FRET experiments, slides were functionalized by incubating the flow cells with 1 mg/ml biotinylated BSA for 5 min and then rinsed. In both experiments, the biotin-presenting surfaces were incubated with 0.1 mg/ml streptavidin solution for 5 min, rinsed and then DNA was added at ∼10 pM for 5 min or until a well-spaced coverage of individual DNA molecules was obtained.

#### Protein–DNA FRET

Cy5-labeled 550 bp T-bulge DNA surfaces were exposed to solutions containing 10 nM AF555-labeled MutS-E315C and 2 mM adenosine diphosphate (ADP) or ATP for imaging as described below.

#### DNA–DNA FRET

Experiments were conducted in two ways: (i) by pre-incubating 20 or 200 nM MutS (unlabeled M88C) and T-bulge DNA with 10 μM ADP, then flowing in 2 mM ADP or ATP, washing out a significant amount of unbound MutS (‘flow’ experiments), and (ii) by injecting 10 or 100 nM MutS (unlabeled M88C or E315C) with 2 mM ADP or ATP into the flow cell containing surface-attached T-bulge DNA. Tubing was attached to drilled holes in the quartz slide to allow for buffer exchange during data acquisition in the ‘flow’ experiments. Because multi-state events are rare relative to the 70% single state events (presumably MutS with ADP bound), we compiled data from multiple experiment days to generate the histograms and transition density plots (TDPs) (Figures 4 and 5; Supplementary Tables S4 and 5). The independent replicate used 10 nM MutS and was conducted by the second method described above (Supplementary Figures S10–13).

At the higher MutS concentrations, we must consider the potential effect of tetramers on our observations (19,49–52). The independent replicate (Supplementary Figures S10–13) was conducted at 10 nM. There is excellent agreement between the FRET values and kinetic rates extracted from both replicates (Supplementary Table S5); therefore, if tetramerization is occurring at the higher concentrations, it has no measurable effect on the observed FRET or kinetics.

### Single-molecule FRET imaging

The samples are imaged using a through-prism total internal reflection fluorescence microscope. Donor and acceptor excitation are achieved using 532 and 640 nm lasers, respectively. The fluorophore emission is collected through a 60X, 1.2 N.A. water immersion objective, and the image is split by a DualView optical splitter with a 645 nm dichroic mirror. The donor and acceptor signals then pass through optical filters before detection by an emCCD camera (585/70 bandpass filter for AF555/TAMRA, 655 longpass filter for Cy5). To observe changes in FRET over time, movies of 800–1000 frames at 100 ms/frame are collected using the following excitation sequence: (i) brief excitation of the acceptor fluorophore (∼1 s) to locate DNA molecules; (ii) excitation of the donor fluorophore (∼2 min) to monitor changes in FRET; (iii) brief excitation of the acceptor fluorophore (∼5 s) to reveal whether the acceptor has photobleached. All experiments are performed at room temperature in 50 mM Tris–HCl pH 7.8, 100 mM sodium acetate, 5 mM magnesium chloride and 2% glucose (w/v), in the presence of an oxygen scavenging and triplet state quenching system of 100 U/ml glucose oxidase, 1000 U/ml catalase, 0.05 mg/ml cyclooctatetraene and 143 mM 2-mercaptoethanol.

### Data analysis

The donor (D) and acceptor (A) signals corresponding to single molecules were detected on separate halves of the emCCD camera and mapped to each other based on images of fixed beads appearing in both channels. Fluorescence intensity versus time traces were generated for the localized donor (I_D_) and acceptor (I_A_) using a custom MATLAB program. The signals were corrected for background and leakage between channels, and the apparent FRET efficiency (E) was calculated as E = I_A_/(I_D_ + I_A_) without gamma corrections (53). Only those time traces exhibiting changes in FRET were analyzed; traces with constant FRET over the entire observation window were not analyzed further for transitions. Only traces with fluorescence intensities consistent with one protein and/or DNA molecule were analyzed. FRET traces showing evidence of protein-dye interactions (i.e. changes in fluorescence intensity of either the donor or acceptor fluorophore that are not anti-correlated) were also discarded from the analysis. Histograms of dwell times were fit to single exponential decays or k_1_k_2_(exp(−k_2_t) – exp(−k_1_t))/(k_1_ – k_2_) to account for a two-step process with two characteristic kinetic rates k_1_ and k_2_. An estimate of error in dwell-time measurements is 0.4 s, which is two measurement bins on each side of the transition. Consequently, states that last <1 s may be missed, which will result in an underestimation of the rate constants (or overestimation of the lifetimes). Previous studies suggest that accurate determination of kinetic lifetimes using the two-step model requires the detection limit to be five times faster than the lifetime (54). In addition, lifetimes shorter than twice the detection limit can have very large error (54). For our experiments, these limits suggest that lifetimes <2 s determined from the two-step model may have large errors.

#### Protein–DNA FRET

MutS binding events were identified based on the criteria described above. Transitions and dwell times for kinetic analysis of AF555-MutS-E315C:Cy5-DNA experiments were extracted from raw FRET efficiency traces by manual identification as described previously (23,26).

#### DNA–DNA FRET

Determination of transitions and kinetics was carried out as described in detail in our recent publication (55). Briefly, a Gaussian derivative kernel (GK) and a modified Chung–Kennedy filter (CK) that takes into account the anti-correlation requirement for FRET (56) were used to detect transitions in FRET efficiency using a custom MATLAB program. We detect transitions using the two independent GK and CK algorithms. After detection of transitions by these two methods, the statistical significance of each transition is determined by a *t*-test and accepted if *P* < 0.05. GK transitions are identified by detecting inflection points in the signal that results from convolving the FRET trace with a GK of various widths. CK transitions are identified by finding local maxima in the standard deviations of forward and backward predictor windows of several data points. Windows that contain transitions have large standard deviations. Taking a weighted average based on their individual confidence scores, we reconcile the timing of transitions identified by the two complementary methods. A separation of at least two data points is required between transitions. Transitions identified by the program undergo final verification by visual inspection. The GK and CK parameters used to analyze these data are outlined in Supplementary Table S3.

Once the transitions were determined, FRET-TACKLE (FRET Transition Analysis Coupled with Kinetic Lifetime Evaluation) analysis was used to extract the pertinent mechanistic information from the compiled data (21,24). With this method, distinct molecular conformations are identified by their characteristic FRET and kinetic lifetime properties. This approach allows molecular states with the same extent of DNA bending (i.e. the same FRET) but distinct kinetics to be distinguished, and *vice versa*. Figures 4 and 5 show the three DNA bending states (Bent States 1–3) in the pathway to MutS mobile clamp formation. Dwell times for Bent States 1 and 3 in the DNA bending experiments were resolved 73 and 69% of the time, respectively. On some occasions, dwell times for Bent States 1 or 3 could not be determined due to the observation window or fluorophore blinking or photobleaching.

## RESULTS

In previous studies, we examined the interaction of MutS with mismatched DNA by monitoring FRET between MutS labeled with a donor at M88C in Domains I and DNA labeled with an acceptor near a T-bulge (unpaired single thymine insertion), in the presence of ADP or ATP (Figure 1). In complementary experiments, we also examined DNA-induced conformational changes in MutS by monitoring intra-protein FRET between donor- and acceptor-labeled Domains I (23,26). The current study expands upon the previous work by focusing on conformational changes in DNA during its interaction with MutS. We take two complementary approaches to monitor DNA dynamics. The first approach employs FRET between AF555-labeled MutS, with the dye located at E315C in Domains III (AF555-MutS-E315), and DNA labeled with an acceptor (Cy5) located 9 bases 3′ from a T-bulge. These experiments are similar to our previous studies with MutS labeled at M88C (23,26); however, M88C is in Domains I which is highly mobile, whereas crystal structures and molecular dynamics simulations suggest that E315 is in a region of the protein that is non-mobile (Figure 1A and Supplementary Figure S2) (17,27,43,57–59). As described below, measuring FRET between AF555-MutS-E315C and Cy5-DNA enables us to monitor DNA conformational changes, while simultaneously following MutS mobile clamp formation. In the second approach, we monitor DNA conformational changes directly by measuring FRET between donor (TAMRA) and acceptor (Cy5) dyes flanking the T-bulge and separated by 19 bp. Aligning the sequence of transitions and comparing the kinetic data from this complementary set of experiments allows us to correlate and characterize in detail the protein and DNA conformational changes that occur during mismatch recognition and subsequent formation of the MutS mobile clamp.

### Protein–DNA FRET senses DNA conformational changes

**Figure 2.**
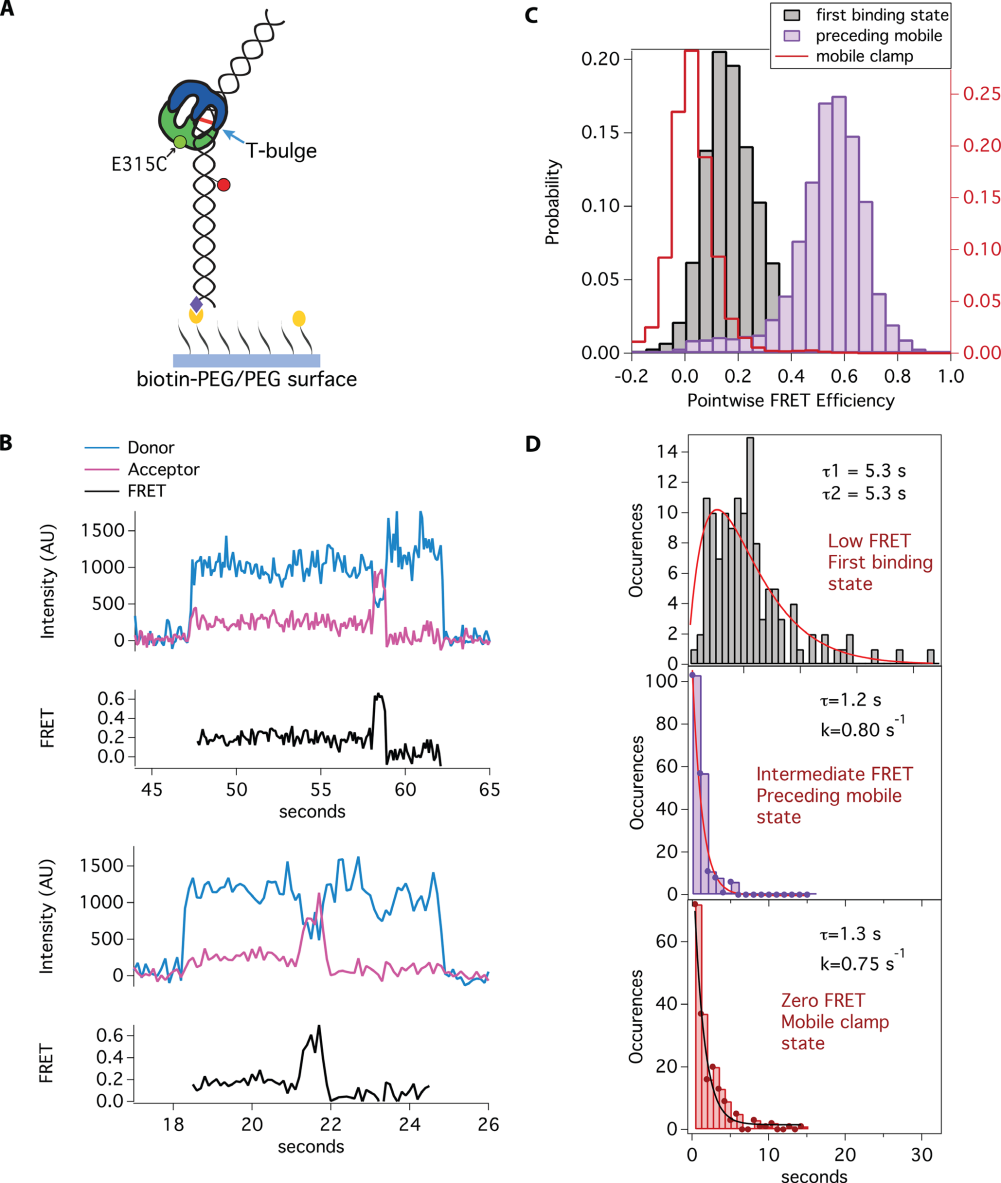
smFRET between AF555-MutS-E315C and Cy5-DNA reveals multistep transition to a mobile clamp. (**A**) Schematic of smFRET experiment with AF555-labeled MutS-E315C and Cy5-labeled T-bulge DNA (550 bp). The mismatch is bound by *Taq* MutS at the single thymine insertion (T-bulge) (blue arrow). The black lines with yellow circles represent biotinylated-PEG and the purple diamonds represent streptavidin. Green and red circles represent AF555 (donor) and Cy5 (acceptor) dyes, respectively. (**B**) Example fluorescence traces in the presence of 2 mM ATP. The blue and magenta traces represent the donor and acceptor signals, respectively, and the black trace shows calculated FRET efficiency. AF555-MutS-E315C is in solution during the entire experiment. There is no fluorescence signal in the traces prior to AF555-MutS-E315C binding mismatch-containing Cy5-DNA (acceptor). Upon MutS binding, donor and acceptor signals are immediately observed, representing FRET between MutS and the DNA. A sequential pathway is observed in the presence of ATP upon MutS binding to the mismatch, then transitioning to the mobile clamp state (low FRET → intermediate FRET → zero FRET). (**C**) Histograms of the distribution of FRET for AF555-MutS-E315C binding to Cy5-DNA in the presence of 2 mM ATP. The first binding state (gray) is low FRET, with the peak centered at 0.15, similar to that observed in the presence of ADP (Supplementary Figure S4B). The second state preceding mobile clamp (purple) exhibits intermediate FRET centered at 0.5, and the final state (red cityscape), before loss of fluorescence, is centered at zero FRET and is consistent with a mobile clamp (23). The fluorescence signal disappears immediately after the protein slides off the free DNA end. (**D**) Plots of the distribution of dwell times for each state fit to one or two exponentials (see ‘Materials and Methods’ section). The dwell-time distribution for the low FRET states (top, gray bars; *n* = 126) fits to two exponentials (τ_1_ = 5.3 s, τ_2_ = 5.3 s, red line), and the dwell-time distributions for the intermediate FRET state (middle, purple bars; *n* = 186), and mobile clamp FRET state (bottom, red bars; *n* = 184) fit to single exponentials with the respective lifetimes of *τ* = 1.2 s and *τ* = 1.3 s. We performed a replicate with a second protein prep (Supplementary Figure S4D). The averages of FRET values and lifetimes for the two replicates are reported in the main text and Supplementary Table S2.

Previously, we monitored the conformational dynamics of MutS as it binds to a mismatch and subsequently forms a mobile clamp by smFRET using a 550-bp DNA substrate with Cy5 located 9 bases 3′ from a T-bulge and *Taq* MutS labeled with AF555 at M88C in the mismatch binding Domain I, which moves upon mobile clamp formation (Figure 1A) (23,26). In the current study (Figure 2A), we performed experiments with MutS labeled at E315C located at the end of the lever domain, which is not expected to be a dynamic region of the protein based on comparisons of different crystal structures and molecular dynamics simulations (17,27,43,57–59) (Supplementary Figure S2). We tested the activity of AF555-MutS-E315C by measuring the binding/bending, unbending and dissociation rates for T-bulge DNA, as well as the ATPase kinetics, using bulk stopped-flow kinetic measurements (Supplementary Figure S3). In DNA unbending/dissociation experiments in the absence of nucleotides, AF555-MutS-E315C exhibits a similar, perhaps marginally reduced, rate (0.04 s^−1^) compared to wild-type (0.06 s^−1^) and unlabeled MutS-E315C (0.05 s^−1^). ATP and ADP accelerate DNA unbending (∼1.5 s^−1^), as reported previously for wild-type MutS, followed by DNA dissociation (0.2 s^−1^) at a slightly slower rate than reported previously for wild-type (0.5 s^−1^) and similar to that of AF555-MutS-M88C (0.2 s^−1^) (23,26,34). Also, AF555-MutS-E315C catalyzes a burst of ATP hydrolysis in the absence of DNA, which is suppressed when it binds T-bulge DNA, again as reported previously for wild-type MutS (16). These data all suggest that the label at E315 may only slightly alter MutS activity.

In the smFRET studies, we observe single step binding and release events (loss of donor and acceptor signal) of AF555-MutS-E315C to the Cy5-T-bulge DNA in the presence of ADP (Supplementary Figure S4A). The distribution of FRET efficiencies fits to a Gaussian centered at 0.21 (Supplementary Figure S4B). The observed FRET of 0.21 is consistent with the distance between dye positions on MutS-E315C and DNA, based on the crystal structure (Supplementary Figure S5) and assuming a Förster radius of ∼50Å–60Å, which is typical for the FRET pairs used in these studies (60). These results are analogous to those from previous experiments with AF555-MutS-M88C, except in that case, the FRET efficiency is 0.65 due to the difference in dye locations (23). The distribution of dwell-times for this state fits well to a single exponential with a lifetime of 4.3 s (Supplementary Figure S4C). This lifetime is slightly longer than that of AF555-MutS-M88C (2.2 s), perhaps due to the label at E315C.

In the presence of ATP, the majority of the T-bulge binding events with AF555-MutS-E315C (71%) are similar to those observed in the presence of ADP, as is the case with MutS-M88C (23). This population represents MutS that has hydrolyzed ATP and retains ADP (16). The remaining 29% of binding events with MutS-E315C exhibit sequential conformational changes to a mobile clamp state (as measured by differences in FRET). Specifically, we observe the transitions: low FRET (0.15) → intermediate FRET (0.5) → zero FRET → no fluorescence signal (Figure 2B and C; Supplementary Table S2). The initial FRET value of 0.15 is slightly lower than the value observed in the presence of ADP (0.21) and represents initial mismatch recognition, while FRET 0.5 represents a conformational change in the MutS-T-bulge complex prior to mobile clamp formation (zero FRET) and dissociation (no fluorescence). (We can distinguish the mobile clamp from dissociation because zero FRET indicates that the donor [MutS] is still on the DNA, but away from the mismatch, and loss of all fluorescence indicates protein dissociation or donor photobleaching.) In previous experiments with AF555-MutS-M88C, we also observed a sequential pattern of transitions, albeit with different FRET values: high FRET (0.65) → intermediate FRET (0.45) → zero FRET (mobile clamp) → no fluorescence signal (protein dissociation or photobleaching). For MutS-M88C, the transition from FRET 0.65 to FRET 0.45 results from a large conformational change in Domains I of the MutS homodimer (23); however, E315C in Domain III is not expected to be very mobile (17,27,57,59), which suggests that the large change in FRET between dyes on MutS-E315C and DNA arises from the dynamics of the DNA in the complex. If so, the structural interpretation would be that DNA straightens out and perhaps rotates such that the label near the T-bulge moves toward the upper channel of MutS (Figure 1A and Supplementary Figure S5). This interpretation is consistent with conclusions from bulk FRET studies on *E. coli* MutS, which suggest that DNA moves toward the upper channel upon mobile clamp formation (58).

Examination of the kinetics of the transitions during mobile clamp formation for MutS-E315C shows that the dwell-time distribution of the first binding state (0.15) exhibits a clear rise and decay (Figure 2D and Supplementary Figure S4D), indicating two rate-limiting steps (61,62) between FRET 0.15 and the next FRET state (0.5), and therefore the existence of two states with FRET 0.15 (designated as 0.15 and 0.15*). Fitting the data with a two-step kinetic model yields lifetimes (τ) of 5.2 and 5.5 s (Supplementary Table S2). These findings parallel the results with MutS-M88C, wherein we also observed two kinetically distinct states in the first binding event, albeit with shorter apparent lifetimes (0.9 and 2.8 s) (Supplementary Table S6). The longer lifetimes for MutS-E315C may result from the label at this position slightly altering the dynamics of the protein. It is worth noting that the lifetimes resulting from these fits to the two-step model can have substantial uncertainty (54) (see ‘Materials and Methods’ section). The distributions of dwell-times for the intermediate state (FRET 0.5) that precedes mobile clamp formation and for the mobile clamp state (FRET 0) both fit well to single-exponential decays with lifetimes of 1.2 and 1.4 s, respectively. These lifetimes are within error of those for MutS-M88C-DNA FRET (FRET 0.45: *τ* = 1.5 s; FRET 0: 2.2 s) and for MutS-M88C intraprotein FRET measurements that reported the relative movements of Domains I in the dimer (FRET 0.65: *τ* = 1.3 s; FRET 0.2 [mobile clamp]: *τ* = 2.4 s) (23). The observation that both M88C and E315C exhibit a specific sequence of three states (resolved by FRET and kinetics) with comparable lifetimes prior to mobile clamp formation strongly suggests that these experiments are reporting on different conformational properties of each of the three states.

### DNA–DNA FRET reveals a previously hidden conformational state

**Figure 3.**
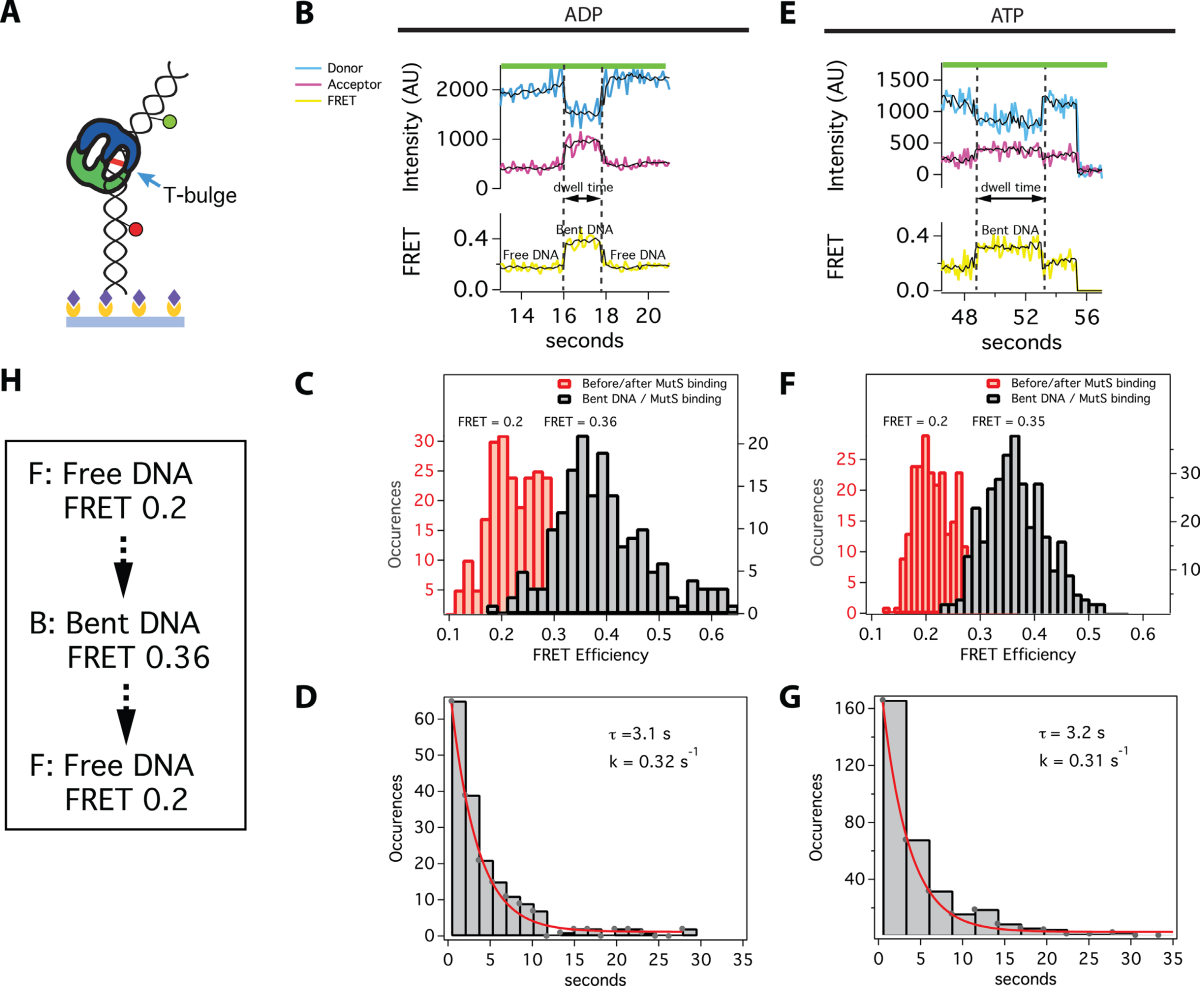
smFRET of MutS-induced DNA bending shows a single bent state for all binding events with ADP and the majority of binding events with ATP (70%). (**A**) Schematic of a surface-attached 68-bp DNA FRET substrate bound by *Taq* MutS at the T-bulge (blue arrow). The yellow circles represent biotinylated-BSA and the purple diamonds represent streptavidin. Green and red circles represent TAMRA (donor) and Cy5 (acceptor) dyes, respectively. Experiments were conducted with 10 nM *Taq* MutS and 2 mM ADP (**B–D**) or with 20–200 nM *Taq* MutS (see ‘Materials and Methods’ section) and 2 mM ATP (**E–G)**. (**B and E**) Example donor (blue) and acceptor (magenta) fluorescence time traces and the corresponding FRET efficiency (yellow). The raw FRET data was smoothed using a modified CK filter (see ‘Materials and Methods’ section) to aid in visualizing the transitions (black lines). Transitions were identified using the raw data. The low FRET and high FRET states are Free DNA (**F**) and Bent DNA (**B**), respectively (see Supplementary Figures S7 and 8 for the homoduplex DNA control and the TDPs). (**C and F**) Histograms of FRET states. (**D and G**) Distribution of dwell-times for high FRET states (Bent DNA) fit to single exponential functions (*n* = 179 for ADP; *n* = 335 for ATP). (**H**) Summary of predominant pathway showing states and FRET values.

To directly monitor the conformation of mismatched DNA upon MutS binding in the presence of ADP and ATP, we measured smFRET using a 68-bp substrate with a T-bulge flanked by TAMRA and Cy5 dyes in the presence of unlabeled MutS (Figure 3A), as done in previous experiments performed in the absence of nucleotides (21,24,63). In the absence of MutS, the DNA exhibits steady FRET with a single peak centered at 0.2 (Supplementary Figures S7C and 8A), which is consistent with the separation between the two fluorophores (19 bp). In the presence of saturating concentrations of ADP (2 mM) and unlabeled MutS, we observe brief anti-correlated changes in the donor and acceptor fluorescence intensities corresponding to increased FRET, which indicates DNA bending (Figure 3B). These events occur only in the presence of MutS and the T-bulge (see homoduplex control in Supplementary Figure S8), and the distribution of FRET states reveals two peaks: one centered at FRET 0.2, which corresponds to free DNA, and the other at FRET 0.36, which corresponds to a single MutS-bound state (Figure 3C). This change in the FRET from 0.2 to 0.36 is similar to that observed with MutS in the absence of nucleotide (21,24) and is consistent with MutS-induced DNA bending as observed in the crystal structures and AFM experiments (17,19,64). Plots of the dwell-time distributions of the bent states (0.36) are fit well by a single exponential, and the lifetime (3.1 s; Figure 3D) is similar to that obtained for protein–DNA FRET for both MutS-M88C (2.3 s) (23) and MutS-E315C (4.3 s; Supplementary Figure S4C), consistent with these different FRET measurements representing the same transition. Taken together, the results indicate that these single FRET events represent ADP-bound MutS binding and bending, and then unbinding from the mismatch (23,26).

**Figure 4.**
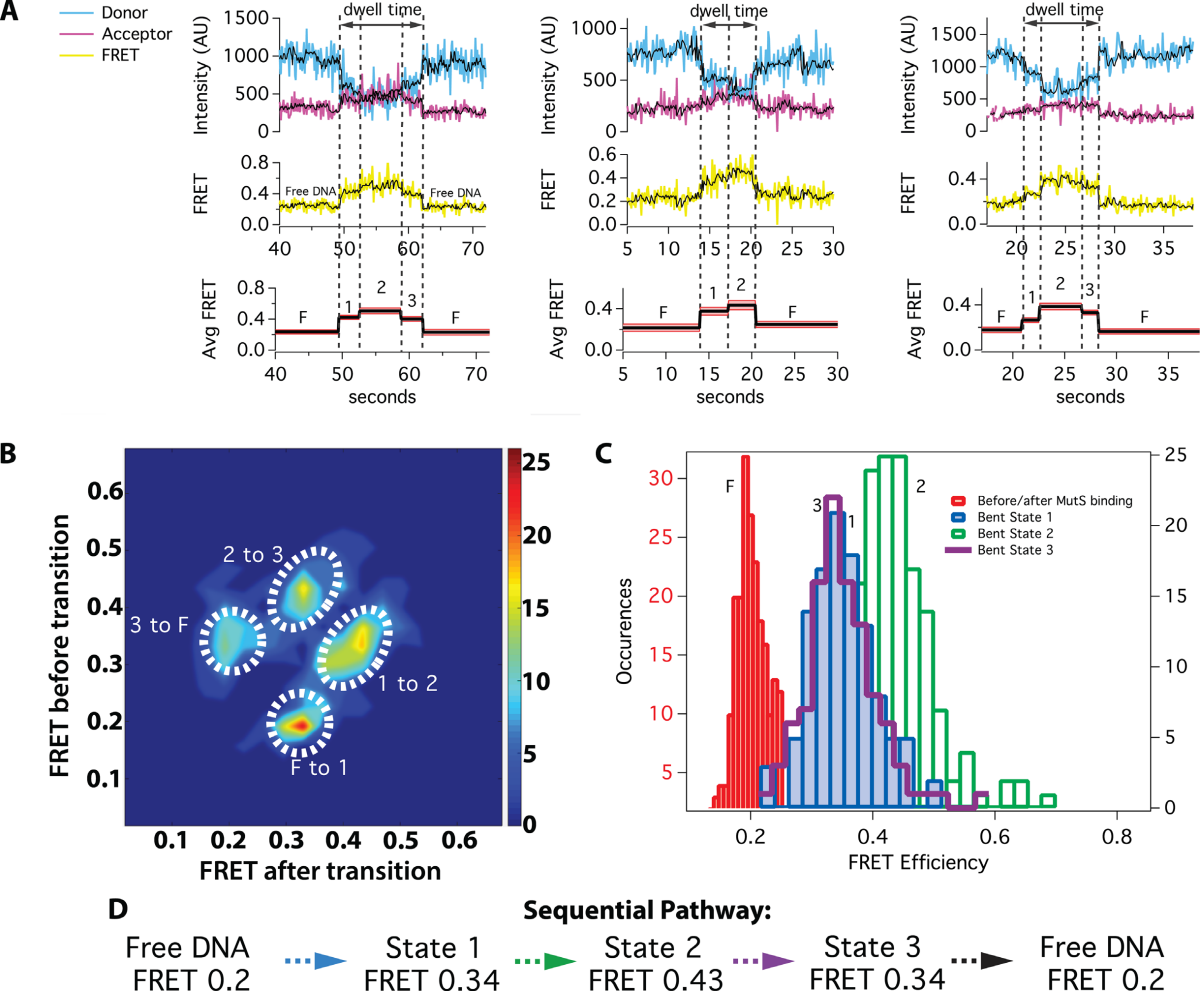
smFRET of DNA bending reveals a pathway of transitions to the mobile clamp for a subset of MutS–DNA complexes with ATP (30%). (**A**) Example donor (blue) and acceptor (magenta) fluorescence time traces (top row) and corresponding FRET efficiency (yellow; middle row) for experiments conducted at 20–200 nM *Taq* MutS (see ‘Materials and Methods’ section) and 2 mM ATP. The black curve shows the data smoothed using a modified CK filter (see ‘Materials and Methods’ section) to aid in visualizing the transitions. Transitions were identified using the raw data. Arrows denote the total time spent in bent states. The bottom row shows average FRET values for each state (black line) and the corresponding standard deviation (red shadow), and the intermediate and high FRET states are numbered 1–3. The low FRET states are labeled Free DNA (F). (**B**) TDP depicting the frequency of transitions between free DNA (F) and Bent states 1 through 3. The states follow a pathway after MutS binds DNA: intermediate FRET (Bent State 1) → higher FRET (Bent State 2) → intermediate FRET (Bent State 3). (**C**) Distributions of FRET values for free DNA (F; red), Bent State 1 (blue), Bent State 2 (green) and Bent State 3 (purple cityscape). (**D**) Summary of predominant pathway showing states and FRET values. The TDP and histograms are generated using data from multiple days. A statistical breakdown of the number of events from each experiment included in the analysis, and the experimental conditions are shown in Supplementary Table S4. In addition, the data from an independent replicate conducted at 10 nM is presented in Supplementary Figures S10–13 and Table S5.

In the presence of ATP (Figure 3E), ∼70% of the bending events mirror those in the presence of ADP, with transitions resulting in a single bent state with FRET 0.35 (Figure 3F), as expected from the protein–DNA FRET experiments. The distribution of dwell times fits to a single exponential function with a lifetime of 3.2 s (Figure 3G). These results are nearly identical to those obtained from DNA–DNA FRET experiments performed in the presence of ADP (Figure 3C and D), indicating that they report ADP-bound MutS binding and unbinding from DNA. In the remaining 30% of MutS-induced DNA bending events detected in the presence of ATP, we observe transitions between more than one bent state (Figure 4A). Most traces exhibit two or three transitions, which do not include the mobile clamp state, because it cannot be differentiated from protein dissociation in DNA–DNA FRET experiments.

**Figure 5.**
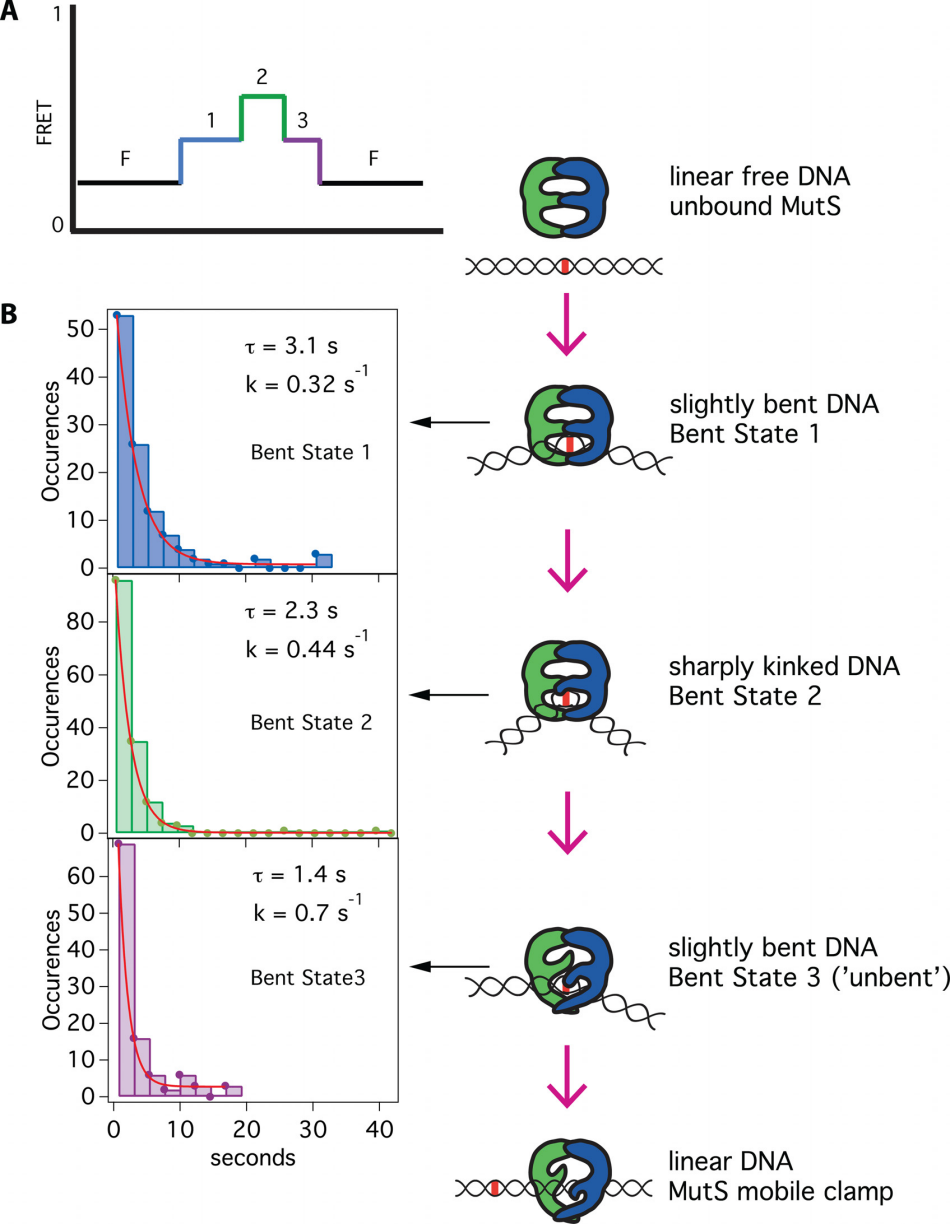
Kinetics of the bent states along the pathway to mobile clamp. (**A**) A schematic of the predominant bending pathway observed in presence of ATP (Figure 4). (**B**) Dwell-time distributions for Bent State 1 (blue bars; *n* = 111), Bent State 2 (green bars; *n* = 153) and Bent State 3 (purple bars; *n* = 105) each fit to single exponential decays (red lines). These individual bent states correspond to the states introduced in Figure 4. Cartoons to the right of the dwell-time distributions suggest conformations associated with each state. Supplementary Figure S9 shows a breakdown of the kinetics for each bent state and TDPs for binding events with 2 and 3 states and for experiments conducted with unlabeled MutS-M88C and MutS-E315C.

Although the changes in FRET efficiency between the different bent states are small, we have developed a rigorous analytical method to identify and validate genuine transitions, which has been described previously in detail (21,55) and outlined in the ‘Materials and Methods’ section. Briefly, we detect transitions using two independent algorithms, a GK and a modified CK filter. After detection of transitions by these two methods, the statistical significance of each transition is determined by a *t*-test and accepted if *P* < 0.05. Computationally determined transitions are verified by visual inspection. To unambiguously determine the transition pathway, the observed changes in FRET are compiled into a TDP (Figure 4B) (21,24). This plot reveals that the majority of bending transitions during mobile clamp formation follow a preferred pathway between three bent states in addition to free DNA (F): from free DNA (low FRET) to an intermediate FRET (Bent State 1; B1), to a slightly higher FRET (Bent State 2; B2), to another intermediate FRET (Bent State 3; B3), back to free DNA (Figures 4 and 5A). Plots of the distribution of FRET for the sequentially observed states reveal single peaks for each state centered at 0.34 for B1, 0.43 for B2 and 0.34 for B3 (Figure 4C and Supplementary Figures S10–13). The distributions of dwell times for B1, B2 and B3 fit to single exponentials with lifetimes of 3.2, 2.3 and 1.4 s, respectively (Figure 5B and Supplementary Figure S12E). The single exponential fits indicate that there are no hidden states in the DNA bending FRET measurements. This result contrasts with the MutS–DNA FRET and MutS-M88C intra-protein FRET measurements, where the dwell-time distributions for the first observed FRET state indicate that there are two states with the same FRET values (FRET 0.15 and 0.15* with MutS-E315C, Figure 2D; 0.65 and 0.65* with MutS-M88C (26); FRET 0.9 and 0.9* with MutS-M88C intra-protein FRET, Supplementary Figure S6). The DNA bending experiments in this study appear to have unmasked the nature of the state hidden in protein-labeled FRET measurements. Notably, in all of these studies, we observe a well-defined sequence of three states prior to mobile clamp formation (note that the mobile clamp cannot be distinguished from dissociation in the DNA–DNA FRET experiments). Correlating these states suggests that B1 and B2 in the DNA bending experiment represent the two kinetically resolved states contained within the first FRET event on the pathway in the protein–DNA and intra-protein FRET studies. In addition, B3 in the DNA bending studies represents the distinguishable FRET state prior to mobile clamp formation in the labeled-protein studies. From here on, we refer to these states as B1, B2 and B3.

**Figure 6.**
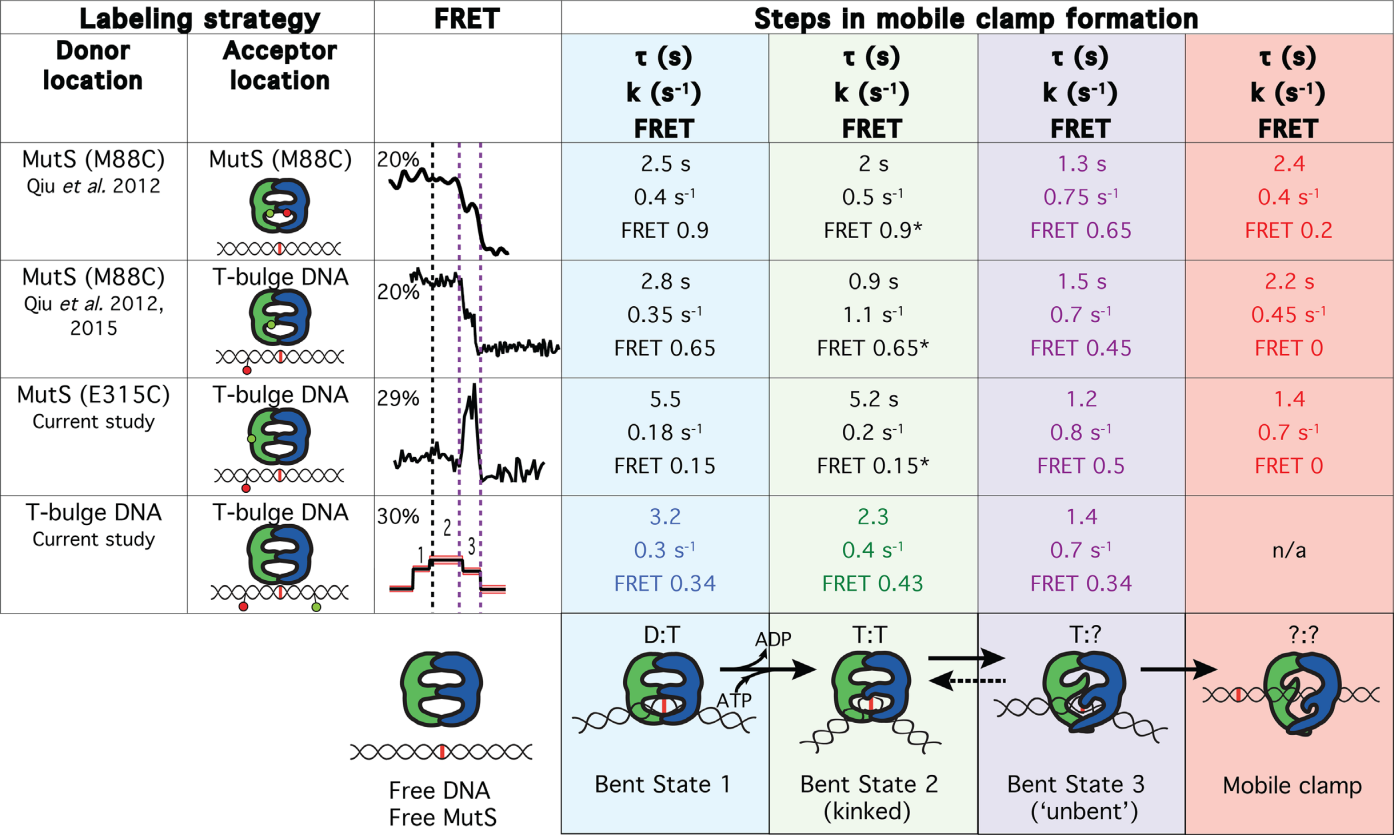
Summary of the FRET and kinetic data on mobile clamp formation and corresponding conformational changes of MutS and DNA. The table summarizes the four different smFRET labeling strategies used to study MMR initiation unified by their transition pathway and kinetics. The conformations of the MutS–DNA complexes at each step are indicated in the bottom row. D and T in the cartoon (bottom row) indicate ADP and ATP, respectively. The locations of the fluorescent labels in the experiments are indicated in the two left columns. An example FRET trace for each experiment is shown in the third column labeled FRET. The dotted lines on the FRET traces denote transitions that were identified both by their position in the pathway and their kinetics. The transition denoted by the dotted black line is only observed by DNA–DNA FRET and corresponds to the hidden transitions in the protein–DNA and protein–protein FRET (identified by fits requiring two kinetic states). The dotted purple lines on the FRET traces indicate transitions that are observed in all FRET signals. Columns 4–7 contain the lifetimes (τ), kinetic rates (k) and FRET values for each state observed by the four FRET labeling strategies (rows). M88C protein–protein FRET measurements (row 2) are a single replicate from a previous publication (indicated column 1). The M88C protein–DNA FRET results (row 3) are the average of three independent experiments (see Supplementary Table S6 for details). Results from the current study (rows 4 and 5) are each the average of two replicates. The lifetime for the second state (column 5) hidden in the first FRET event in protein-labeled FRET, but kinetically resolved from the two-step fitting function (rows 2–4), is sensitive to detection efficiency of the shortest events. We estimate our uncertainty in this lifetime from comparison of multiple replicates to be ∼2 s under our current experimental conditions (see ‘Materials and Methods’ section). Supplementary Table S6 shows the kinetic constants and their 95% confidence intervals for each experiment and the average and standard deviation for the replicates.

The consistency among the kinetics for these states measured by the different FRET reporters supports their identification as the states on a three-step sequential pathway. The lifetimes of B3, which is the best-determined state, measured by all the different experiments are within 10% of one another (average 1.35 s; standard deviation 0.1 s). With the exception of E315C, the lifetimes of B1 are all within 15% of the mean (average 2.8 s; standard deviation 0.4 s). The lifetime of B1 for E315C is significantly longer (5.5 s), which we attribute to the impact of a fluorophore at this label site. The lifetimes for B2 have the largest variation among experiments (ranging from ∼1 to 5 s); however, these lifetimes are the most difficult to determine accurately. Specifically, this parameter results from fitting to the two-step kinetic model, and it is highly sensitive to missed events with short duration, near our detection limits (54). Notably, the B2 lifetime determined from the DNA bending FRET experiments (2.3 s) is near the middle of the range of the lifetimes determined from the labeled-protein FRET experiments. Taken together, these results strongly suggest that the three bending states (B1, B2 and B3) correspond to states seen in the MutS–DNA and intra-protein FRET experiments (Figure 6).

## DISCUSSION

To initiate MMR, MutS must first locate and recognize a mismatch. After mismatch recognition, ATP induces conformational changes in the MutS–DNA complex such that MutS can convert to a mobile clamp state and/or interact with MutL to trigger the cascade of events that leads to DNA repair (3). In previous smFRET studies, we monitored the interaction of MutS with mismatched DNA and the associated conformational changes in DNA-binding Domains I of MutS (23,26). The findings revealed that after mismatch recognition, MutS converts into a mobile clamp via two sequential conformational transitions that are accompanied by large movements of Domains I. In the current study, we expanded our smFRET repertoire to monitor corresponding conformational changes in DNA during the same ATP-dependent transitions (Figure 1B).

Our measurements of ATP-dependent MutS-induced DNA bending reveal the following pathway: Free DNA (FRET 0.2) → Bent State 1 (FRET 0.34) → Bent State 2 (FRET 0.43) → Bent State 3 (FRET 0.34) → mobile clamp and/or free DNA (FRET 0.2) (Figure 4). Similar dominant bending transitions were observed in the absence of nucleotides in our earlier studies (21,24), which led us to suggest that conversion of DNA to an unbent (or slightly bent) conformation was a necessary precursor to formation of the MutS mobile clamp (19–21,24,65). The current results support and extend this conclusion.

By comparing FRET transitions and kinetic data from complementary measurements utilizing four different strategies (Figure 1B), we have been able to identify coordinated conformational changes in both MutS and DNA as MutS transitions from initial mismatch recognition to a mobile clamp state (Figure 6). These transitions are linked to changes in ADP and ATP occupancies of the MutS dimer, as suggested in previous studies (15,16,39). Homodimeric *Taq* MutS contains two ATPase sites that communicate with one another, resulting in asymmetric ATP binding and hydrolysis in the two sites (15,16,39). In the absence of DNA, one site has high affinity for ATP and the other for ADP (ATP:ADP liganded), although the overall ATPase cycle can produce transient states with varying nucleotide occupancies, including ATP:ATP, ADP:ADP, empty:ADP and empty:ATP (15). Our data, together with the previous structural and functional studies on MutS DNA binding and ATPase activities (15,23,26,66–70) as well as MutS and DNA dynamics (19–21,23,24,26), allow us to propose a detailed model of MutS and DNA conformational changes that govern mismatch recognition and lead to MutL recruitment and mobile clamp formation.

In the absence of DNA, MutS exists mainly in two conformations: one with Domains I open (MutS-M88C intra-protein FRET 0.2) and the other with Domains I closed (MutS-M88C intra-protein FRET 0.9) (23,26). During the one-dimensional search for a mismatch, Domains I close to interact with DNA (23). This closure appears to ‘push’ on the DNA to induce bending, which is facilitated electrostatically by the conserved Glu of the Phe-Xaa-Glu motif in Domains I (19,20). Upon mismatch recognition, Phe and Glu from one MutS subunit make specific contacts with one base of the mismatched pair, and the DNA transitions from a smooth bend to a kink at the mismatch (19,20), as observed in the crystal structures (17,18,27–29,64). This state corresponds to B1 (Figure 6), and is similar to states we have observed previously without nucleotides (21,24). In the absence of nucleotides, MutS can remain at the mismatch for long periods (>30 s), visiting multiple bent states (19,21,24). When MutS is doubly-liganded with ADP, MutS stays at the T-bulge for only 2–4 s and induces a single bent state that is indistinguishable from B1 (Figures 3C and 4C). MutS–DNA complexes that contain one ATP and one ADP can progress from B1 through an ordered sequence of states leading to formation of a mobile clamp (23,26). After initial recognition (B1), the MutS–T-bulge complex undergoes two additional conformational transitions prior to mobile clamp formation, one of which is expected to be associated with ADP-ATP exchange to form an ATP:ATP liganded state (71). The first transition after recognition results in additional DNA bending at the mismatch (B2), with Domains I maintaining a similar position (0.9 in MutS-M88C intra-protein FRET for both B1 and B2). The increase in bending likely results from slight Domain I movements that push further on the DNA. This idea is consistent with subtle differences in the positions of Domains I seen in crystal structures of a MutS–T-bulge complex liganded with either ADP or the ATP transition state analog ADP-beryllium fluoride (18,64). Subsequently, this state (B2) transitions to a state that precedes the mobile clamp (B2 to B3) through coordinated conformational changes in both DNA and MutS Domains I. Specifically, Domains I separate (i) and the DNA begins to unbend (ii), moving away from Domains I (iii) and toward the upper channel closer to lever Domains III (iv) (i: M88C-M88C intra-protein FRET 0.9* to 0.65; ii: DNA FRET 0.43 to 0.34; iii: M88C-DNA FRET 0.65* to 0.45; iv: MutS-E315C-DNA FRET 0.15* to 0.5). At this point, MutS can recruit MutL to the mismatch or convert to a mobile clamp (26). Together, the data suggest that DNA unbending and the associated opening of Domains I to form B3 is critical for activating the downstream events that lead to repair. Consistent with this idea, statically bent palindromic mispairs (72) are bound tightly by MutS, but the complex does not recruit MutL *in vitro* and such mispairs are refractory to repair *in vivo* (19,73–75). Accordingly, we posit that Bent State 3 is a central regulatory element governing MMR, because it is required for both conversion of MutS to a mobile clamp and for recruitment of MutL to initiate repair.

Insight into the structural features of this state can be gleaned from a recent crystal structure of the *E. coli* MutS–MutL complex containing two non-hydrolyzable ATP analogs (58). Although Domains I are disordered in this structure, the adjacent connector Domains II are ordered, and there is a large shift in their positions relative to the *E. coli* MutS–DNA–ADP structure (17,27). The relocated connector domains interact with the ATPase Domains V and expose part of the MutL binding surface on MutS. Our finding that MutL can interact with B3 while MutS remains at the mismatch (26) suggests that Domain I of subunit A (MSH6, eukaryotic homolog) remains engaged with the mismatch while Domain I of subunit B (MSH2, eukaryotic homolog) moves up toward the ATPase Domains, such that the connector Domain can interact with MutL. We speculate that movement of subunit B Domain I may be driven, in part, by the straightening of DNA during the transition from B2 to B3. Our previous studies (23) suggest that the transition from B1 to B2 is associated with ADP-ATP exchange, and it is tempting to speculate that the transition from B2 to B3 may be coupled with changes in nucleotide occupancy. Our data showing that Domains I oscillate between open and closed in the mobile clamp state (23) further raise the possibility that the diffusively-translocating MutS mobile clamp (14) could continue to cycle through ATP hydrolysis and ADP release. These details advance our understanding of how coordinated structural transitions of mismatch DNA and MutS are coupled to nucleotide occupancy at the asymmetric ATPase sites on MutS.

The ordered sequence of events in the MutS–mismatch complex leading to MutL recruitment and the mobile clamp formation also suggests a push-me/push-back model for the energetics of MutS–DNA interactions. In this model, MutS bends DNA at a mismatch upon initial recognition; ADP-ATP exchange in MutS results in MutS pushing further on the DNA to increase bending (B2); the increased stress in DNA is released by DNA unbending (B3) and pushing back on the protein to partially open Domains I, perhaps aided by changes in nucleotide occupancy, which in turn drives conformational transitions in MutS toward formation of a mobile clamp. The energy from DNA unbending in the B2 to B3 transition may be sufficient to drive the release of MutS Domains I from the mismatch, providing a plausible explanation for the observation that mobile clamp formation can occur in the presence of non-hydrolyzable ATP analogs (14,22,23). This push-me/push-back model intimates that local DNA flexibility plays a central role in mismatch verification and impacts whether a given DNA site bound by MutS will go on to be repaired (31), and it provides a structural framework for understanding differences in repair efficiencies based on the type of mismatch or its sequence context (76–80). If the DNA is too stiff for MutS to induce bending, then MutS will not be able to recognize the mismatch. Alternatively, if the DNA is statically bent and cannot undergo the unbending transition to Bent State 3, MutS cannot recruit MutL to complete repair. In summary, our experiments correlating conformational changes in the protein and DNA reveal key structural transitions and driving forces that lead to the initiation of DNA MMR. Applying this correlational approach in future investigations should yield deeper insights into why repair-deficient mismatches and MutS mutants disrupt initiation of MMR.

## Supplementary Material

Supplementary Data
