# Supplementary material for: Coordinated protein and DNA conformational changes govern mismatch repair initiation by MutS
Source: Nucleic Acids Res. 2018 Oct 1;46(20):10782–95. doi: 10.1093/nar/gky865 (PMC6237781; doi:10.1093/nar/gky865)
Supplement: Supplementary Data [file gky865_supplemental_files.pdf]

Coordinated protein and DNA conformational changes govern mismatch repair initiation by MutS

SUPPLEMENTARY DATA

Sharonda J. LeBlanc†, Jacob W. Gauer†, Pengyu Hao, Brandon C. Case, Manju M. Hingorani, Keith R. Weninger, and Dorothy A. Erie

†These authors contributed equally to this work.

Table of contents

I. Determination of labeling efficiency
II. 68-bp DNA substrate
III. E315C label site on MutS
  a. Crystal structures
  b. Ensemble data
IV. Protein-DNA smFRET experiments
  a. E315C
  b. Dye mobility simulations
  c. M88C
V. DNA-DNA smFRET experiments
  a. ADP
  b. Statistics
  c. ATP

I. Determination of labeling efficiency

Free AlexaFluor 555 (AF555) dye was removed from the labeled protein using a gel filtration column. The labeling efficiency was estimated by analyzing a UV/Vis spectrum of the AF555-MutS protein solution. We used the following parameters to calculate the concentrations of protein and dye from the Beer-Lambert law ($A = \varepsilon C \ell$, where ℓ is the cell path length = 1 cm):

Supplementary Table 1: Dye labeling efficiency parameters

	Absorbance (A)	Extinction Coefficient (ε)	Concentration (C)
MutS	280 nm absorbance (A_{280}, corrected for dye absorbance at 280 nm)<br><br>= 0.06	52,720 $M^{-1}\,cm^{-1}$ (for the monomer)<br>Source: calculated using ExPASy ProtParam tool based on the PDB sequence	1.1 µM
AF555	555 nm absorbance (A_{555})<br><br>= 0.15	150,000 $M^{-1}\,cm^{-1}$<br>Source: Thermo Fisher Scientific	1 µM

The dye labeling efficiency was calculated as C_{AF555}/C_{MutS}= 1/1.1 = 0.91 = 90 %

II. 68-bp DNA substrate

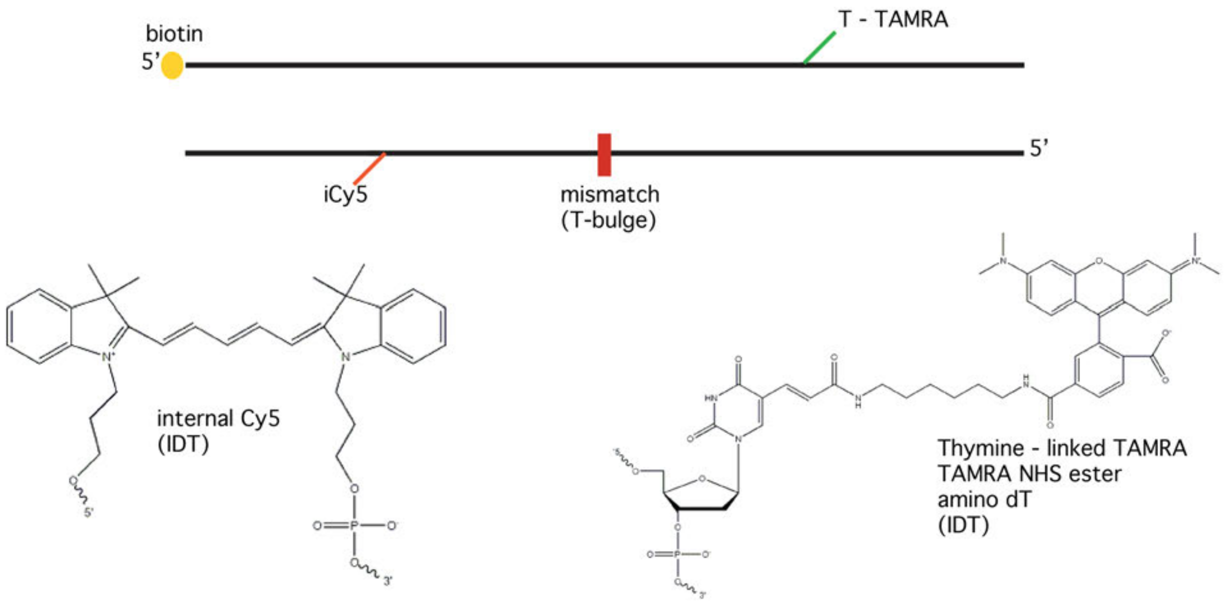

Figure S1: A graphical representation of the 68-bp TAMRA-Cy5-labeled duplex DNA used in DNA-DNA FRET experiments (Figures 3-5 in the main text and supplementary section V).

III. E315C label site on MutS

<u>III.a. Crystal structures</u>

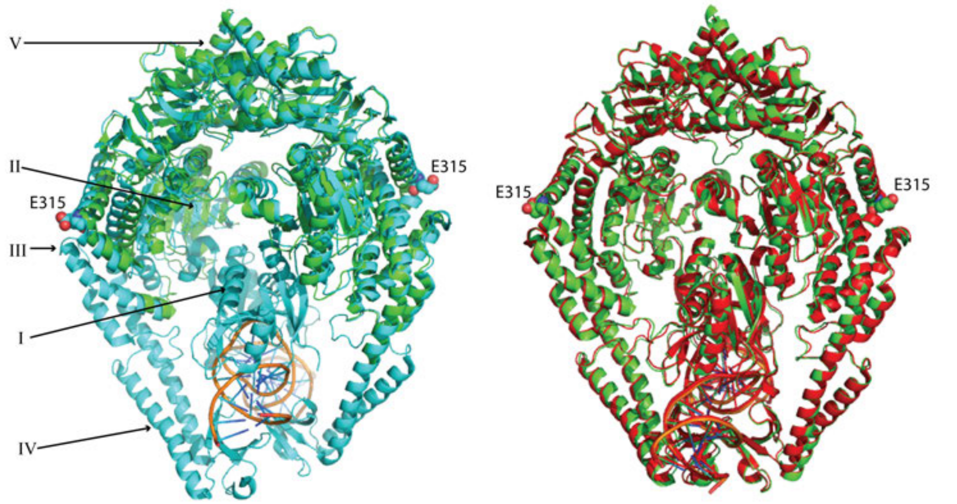

Figure S2: (LEFT) Comparison of of *Taq* MutS without DNA (PDB ID: 1EWR (1)) and with DNA (PDB ID: 1EWQ (2)). An overlay of *Taq* MutS crystal structures without DNA (green) and with DNA (blue) shows that residue E315 maintains its position and the surrounding portion of the lever Domain III is structurally stable, supporting the idea that a conformational change in DNA is responsible for the change in FRET (0.15 → 0.5) observed in AF555-MutS-E315C:Cy5-DNA experiments (Figure 2 in the main text.). Note that in the structure without DNA, Domains I and IV are too mobile to be resolved, indicating that unlike E315, residue M88 in Domain I utilized in previous studies (3,4) changes position upon MutS binding to DNA. **(RIGHT) Comparison of *Taq* MutS-DNA without nucleotides (PDB ID: 1EWQ, red) and with ADP-beryllium trifluoride (PDB ID:1NNE (5), green).** An overlay of nucleotide-free/bound MutS structures also shows that residue E315 is in a relatively non-mobile part of the MutS protein.

III.b. Ensemble data

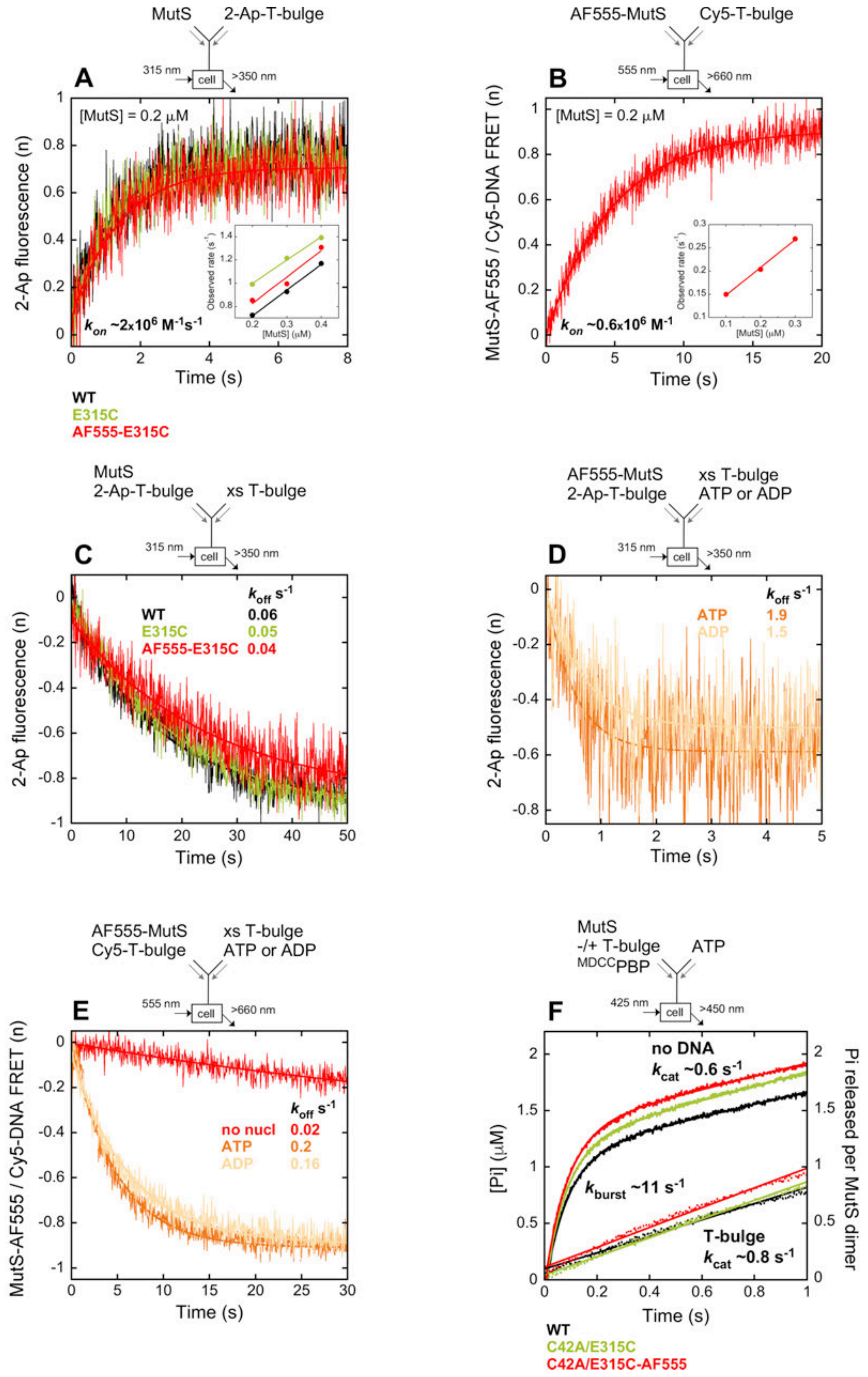

Figure S3: Native MutS-like mismatch binding and ATPase kinetics of Alexa Fluor 555-labeled MutS-E315C. DNA binding properties of wild type and unlabeled or labeled MutS-E315C were compared using a 37 bp DNA substrate with 2-aminopurine (2-Ap) positioned adjacent to a T-bulge, as described previously (6,7). **A**) Mismatch binding kinetics were measured by mixing MutS with 2-Ap-T-bulge DNA in a KinTek stopped-flow instrument and monitoring the increase in 2-Ap fluorescence over time due to DNA bending (final concentrations: 0.2-0.4 μM MutS dimer, 30 nM 2-Ap-T-bulge, 50 mM Tris-HCl, pH 7.5, 100 mM sodium acetate, 5 mM $MgCl_2$, 40 ºC); kinetic trace shown at 0.2 μM protein. The signal from 3 or more traces was averaged for each experiment, corrected for MutS background fluorescence, normalized and fit to a single exponential function. A linear dependence of the DNA binding/bending rate on MutS concentration (inset) yields bimolecular binding rate constants of 2.2, 2 and 2.3 x 10^6 M^{-1} s^{-1} for wild type, MutS-E315C and AF555-MutS-E315C, respectively. **B**) The new reporter assay developed in this study, FRET between AF555 (donor) on E315C and Cy5 (acceptor) located 9 bp from the T-bulge on a 69 bp DNA substrate (6,7), also yields a similar mismatch binding rate of 0.6 x 10^6 M^{-1} s^{-1} in analogous measurements. In this case, increase in Cy5 fluorescence upon mixing AF555-MutS-E315C with Cy5-T-bulge in the stopped-flow was monitored over time (final concentrations: 0.1-0.3 μM AF555-MutS-E315C dimer, 40 nM Cy5-T-bulge, 50 mM Tris-HCl, pH 7.5, 100 mM sodium acetate, 5 mM $MgCl_2$, 40 ºC); kinetic trace shown at 0.2 μM protein and concentration dependence of the rate shown in inset. **C**) DNA unbending kinetics were measured first in the absence of nucleotides by mixing MutS and 2-Ap-T-bulge DNA with excess unlabeled T-bulge DNA chase and monitoring decrease in 2-Ap fluorescence over time due to DNA unbending, which is followed by release from MutS (final concentrations: 0.2 μM MutS dimer, 30 nM 2-Ap DNA, 8 μM unlabeled T-bulge, 50 mM Tris-HCl, pH 7.5, 100 mM sodium acetate, 5 mM $MgCl_2$, 40 ºC). The signal from 3 or more traces was averaged for each experiment, corrected for MutS background fluorescence, normalized and fit to a single exponential function. The rates were 0.06, 0.05 and 0.04 s^{-1} for wild type, MutS-E315C and AF555-MutS-E315C, respectively, similar to previous reports (6,7). The k_{off}/k_{on} ratios yield K_d values of 30, 26 and 17 nM for the three proteins, respectively, also in line with previous reports (6,7). **D**) The effects of nucleotides on the AF555-MutS-E315C-T-bulge complex were also measured for comparison with wild type MutS. On mixing AF555-MutS-E315C and 2-Ap-T-bulge DNA with excess unlabeled T-bulge DNA and either ATP or ADP in the stopped flow, 2-Ap fluorescence decreases over time due to DNA unbending, which is followed by DNA release (final concentrations: 0.2 μM MutS dimer, 30 nM 2-Ap-T-bulge, 8 μM unlabeled T-bulge, 0.5 mM ATP or ADP, 50 mM Tris-HCl, pH 7.5, 100 mM sodium acetate, 5 mM $MgCl_2$, 40 ºC). The signal from 3 or more traces was averaged for each experiment, corrected for MutS background fluorescence, normalized and fit to a single exponential function. ATP and ADP accelerate DNA unbending to 1.9 and 1.5 s^{-1}, respectively, as reported previously for wild type MutS (6,7). **E**) DNA dissociation kinetics were measured by mixing AF555-MutS-E315C and Cy5-T-bulge with excess unlabeled T-bulge DNA in the absence of nucleotides or with ATP or ADP in the stopped flow, and monitoring decrease in Cy5 acceptor fluorescence over time (final concentrations: 0.1 μM MutS dimer, 40 nM Cy5-T-bulge, 2 μM unlabeled T-bulge, 0.5 mM ATP or ADP, 50 mM Tris-HCl, pH 7.5, 100 mM sodium acetate, 5 mM $MgCl_2$, 40 ºC). The signal from 3 or more traces was averaged for each experiment, corrected for MutS background fluorescence, normalized and fit to a single exponential function. The rates were 0.02, 0.2 and 0.16 s^{-1} without nucleotide, with ATP and with ADP, respectively, comparable to previously reported rates (6,7). **F)** ATP hydrolysis and phosphate (Pi) release rates were measured for wild type and unlabeled or labeled MutS-E315C in the absence or presence of a 37 bp T-bulge DNA using a Pi-binding MDCC-PBP reporter assay, as described previously (6,8). Mixing MutS with ATP in the stopped-flow in the absence of DNA results in a burst of ATP hydrolysis and Pi release followed by a linear steady state phase (final concentrations: 1 μM MutS dimer, 1 mM ATP, 15 μM MDCC-PBP, 50 mM Tris-HCl, pH 7.5, 100 mM sodium acetate, 5 mM $MgCl_2$, 40 ºC). The signal from 3 or more traces was averaged for each experiment, converted to Pi concentration using a calibration standard, and fit to an exponential + linear function. Wild type MutS rapidly hydrolyzes 1 ATP/dimer at 11.5 ± 0.1 s^{-1} followed by a slower k_{cat} of 0.6 s^{-1} (slope/[MutS]). MutS-E315C and AF555-MutS-E315C exhibit similar burst and steady state rates of 11.9 ± 0.1 s^{-1}, 0.6 s^{-1} and 11.4 ± 0.1 s^{-1}, 0.6 s^{-1}, respectively. Preincubating MutS with T-bulge DNA suppresses burst the ATPase activity (final concentration: 3 μM T-bulge), as reported previously (6,8), and all three proteins exhibit ATPase rates of ~0.7 - 0.9 s^{-1}.

Note: In the ensemble experiments, MutS is pre-incubated with mismatch DNA in the absence of nucleotide, and then ATP is added, resulting in essentially a single population of starting states. In

contrast, the single molecule experiments are under steady state conditions in which MutS, DNA and ATP are present simultaneously resulting in multiple populations of nucleotide-bound MutS, which are absent in early time points in ensemble experiments.

IV. Protein-DNA smFRET experiments

IV.a. E315C

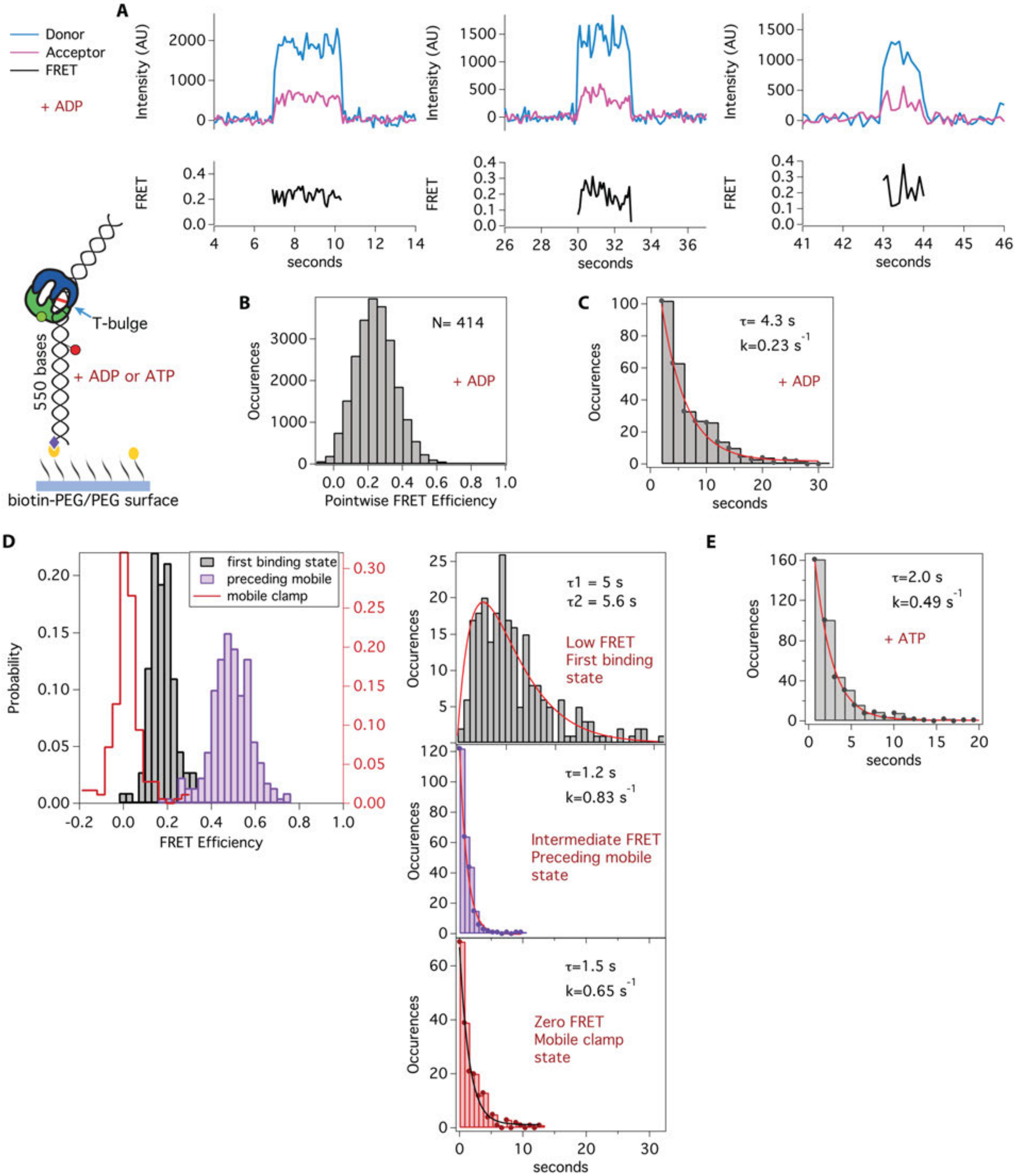

Figure S4: **Results of Alexa Fluor 555-MutS-E315C binding to Cy5 DNA (550-bp T-bulge) in the presence of ADP or ATP. A)** Representative traces of single MutS-DNA bind and release events in the presence of ADP and 10 nM AF555-MutS-E315C (Blue = donor signal; magenta = acceptor signal; black = FRET). **B)** A histogram of the calculated FRET when AF555-MutS-E315C binds to Cy5-DNA in the presence of ADP centers on a low FRET value of 0.21. In our previous AF555-MutS-M88C experiments, MutS bound to the T-bulge in a single high FRET state (0.65). The 0.65 FRET value is consistent with

closer proximity of M88 to the mismatch (and thus Cy5 label) than E315, which is farther from the mismatch and thus yields a lower FRET value upon MutS binding. **C)** The kinetics of AF555-MutS-E315C binding in the presence of ADP exhibits single exponential behavior with a lifetime of 4.3 s (n = 414). **D)** In the presence of ATP and 10 nM AF555-MutS-E315C, a sequential pathway of FRET states is observed that represents MutS binding to the mismatch, then transitioning to the mobile clamp state (low FRET → intermediate FRET → zero FRET). The first binding state (gray) is low FRET, with the peak centered at 0.15, similar to that observed in the presence of ADP. The second state preceding mobile clamp (purple) exhibits intermediate FRET centered at 0.47, and the final state (red cityscape), before loss of fluorescence, is centered at zero FRET and is consistent with a mobile clamp. Plots of the distribution of dwell times for each state fit to one or two exponentials (see Materials and Methods in the main text). The dwell-time distribution for the low FRET states (top, gray bars; n = 231) fits to two exponentials ($\tau 1$ = 5 s, $\tau 2$ = 5.6 s, red line), and the dwell-time distributions for the intermediate FRET state (middle, purple bars; n = 261), and mobile clamp FRET state (bottom, red bars; n = 192) fit to single exponentials with the respective rate constants of τ = 1.2 s and τ = 1.5 s. This is a replicate of data presented in Figure 2 of main text. The FRET values and kinetic rates reported in the main text are averages of the two replicates (see Supplementary Table 2). **E)** For 71% of AF555-MutS-E315C binding events in the presence of ATP, we observe a single FRET state as with ADP. The kinetics exhibit single exponential behavior with a lifetime of 2.0 s (n = 393), which is nearly identical to the lifetime that we measured for AF555-MutS-M88C in the presence of ATP (2.22 s)(3)

Supplementary Table 2: Summary of protein-DNA smFRET replicate experiments with AF555-MutS-E315C binding to 550bp Cy5-DNA.

**FRET state**	**Replicate 1[§]**			**Replicate 2***			**Avg. ± Standard Deviation**		
	FRET	k (s^{-1})	τ (s)	FRET	k (s^{-1})	τ (s)	FRET	k (s^{-1})	τ (s)
First binding	0.14	0.19<br>0.19	5.3<br>5.3	0.15	0.2<br>0.18	5.0<br>5.6	**0.15** ± 0.01	**0.2** ± 0.01<br>**0.19** ± 0.01	**5.2** ± 0.2<br>**5.5** ± 0.2
Intermediate	0.53	0.8	1.2	0.47	0.83	1.2	**0.5** ± 0.04	**0.82** ± 0.02	**1.2** ± 0.1
Mobile clamp	0.0	0.75	1.3	0.0	0.65	1.5	**0.0** ± 0.0	**0.7** ± 0.07	**1.4** ± 0.2

[§]Presented in Figure 2 of main text.
*Second protein prep, combined data from experiments conducted on 2 different days.

IV.b. Dye mobility simulations

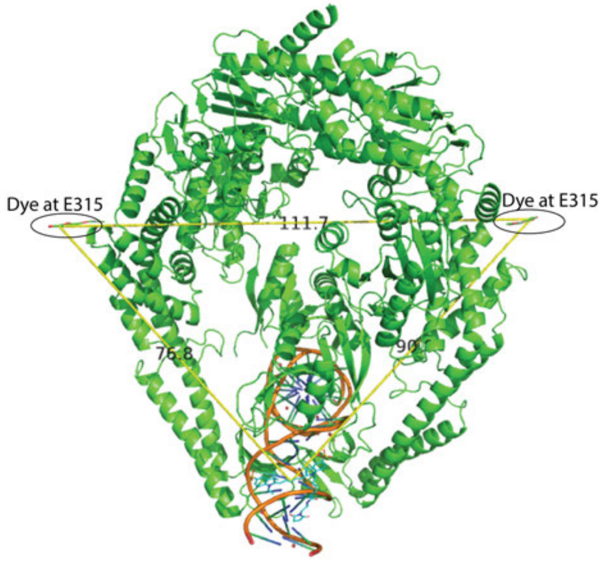

In the AF555-MutS-E315C experiments, DNA unbending (Figures 2, 4 and 5 in the main text; Bent State 3) would position the acceptor dye on DNA closer to the donor on E315C, resulting in the observed intermediate FRET value (0.5). To test this idea, we made some simple structural models of the MutS-DNA complex, which revealed a decrease in distance between AF555-E315C and Cy5-DNA when DNA is straightened (‘unbent’) in the complex (Figure S5). Thus, we attribute the observed change in FRET (0.15 → 0.5) to a conformational change in DNA. The large conformational change in the MutS DNA binding Domains I identified in previous experiments (3) is unlikely for the E315C site.

Figure S5: Structural model of the MutS-DNA complex with attached dyes. Molecular dynamics

were used to determine the average position of Alexa Fluor 555 dye molecules at E315C sites on the *Taq* MutS homodimer (PDB ID:1EWQ) (modeled as Cy3; see Materials and Methods in the main text). The labeled distances in Figure S5 are in Angstroms (Å). The distance between the two dyes is ~110 Å, which precludes intra-protein FRET as was observed with M88C (3). The distance between each donor dye at E315C and acceptor Cy5 on DNA (cyan) was also measured. Bent DNA (~60°), as shown in the crystal structure, yields two possible distances between donor and acceptor of 77 Å and 90 Å. The average distance between AF555-E315C (on MutS) and Cy5 (on DNA) is ~80 Å. Straightening ('unbending') the DNA results in a donor-acceptor average distance of ~70 Å, consistent with the observed transition to the intermediate FRET value (0.15 → 0.5) in Figure 2 of the main text. PDB files for models are available upon reasonable request.

IV.c. M88C

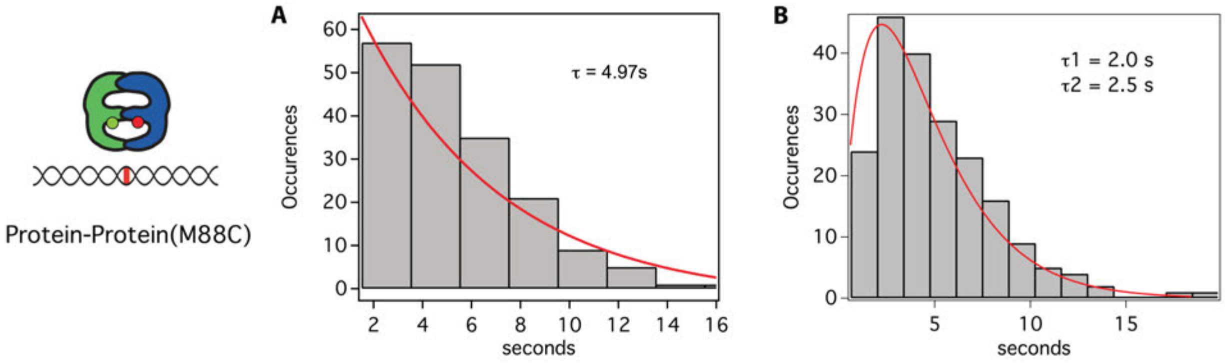

Figure S6: The kinetics of donor-acceptor-MutS-M88C binding to 550-bp T-bulge DNA (first binding state). In previous studies, we determined the kinetics of MutS conformational changes upon binding to unlabeled T-bulge DNA in the presence of ATP. We observed a large conformational change in the DNA binding Domains I (containing M88C) indicated by a decrease in FRET (0.9 → 0.65) just prior to the mobile clamp state (FRET 0.2) (Figure 6 in the main text). **A)** Previously, we fit the dwell time histogram of the first binding state to a single exponential function (τ = 4.97 s) (3) **B)** Re-binning the dwell-time data plotted in **A** (from ref (3)) showed a clear rise and decay in the distribution, indicative of two kinetic states involved in the first binding event (τ1 = 2.0, τ2 = 2.5, n = 200), consistent with subsequent experiments (4) and the current study (Figure 2D in the main text). We also observed these two previously unresolved states directly with DNA-DNA FRET experiments (Figures 4 and 5 in the main text and supplementary section V).

V. DNA-DNA smFRET experiments

V.a. ADP

We determined the DNA bending properties of *Taq* MutS in the presence of saturating concentrations of ADP and ATP, by measuring the fluorescence intensities of donor and acceptor dyes flanking a T-bulge and calculating FRET efficiency as a function of time (Figure 3 in the main text and Figure S7). For the 68-bp DNA substrate, the FRET efficiency remained constant over time with a peak centered on 0.2 in the absence of MutS and nucleotide (Figure S7C, black dotted cityscape). Upon addition of 10 nM *Taq* MutS and 2 mM ADP, anti-correlated changes in donor and acceptor fluorescence intensity traces were observed, resulting in brief periods of increased FRET efficiency (0.36) followed by a return to the original FRET efficiency (0.2). In the transition density plot, the FRET efficiency was found to transition between only two distinct FRET states, low and high, which correspond to Free DNA (F) and Bent DNA (B), respectively. *Taq* MutS bends DNA to a single bent state (B) in the presence of ADP. For ~70% of MutS binding events in the presence of ATP, only a single bent state is observed that is very similar to the ADP single bent state. The other 30% of binding events exhibit a sequential pathway of bent states that correlates with MutS mobile clamp formation (3,4) (Figures 4-6 in the main text and Figures S10-13 for independent replicates in the presence of ATP).

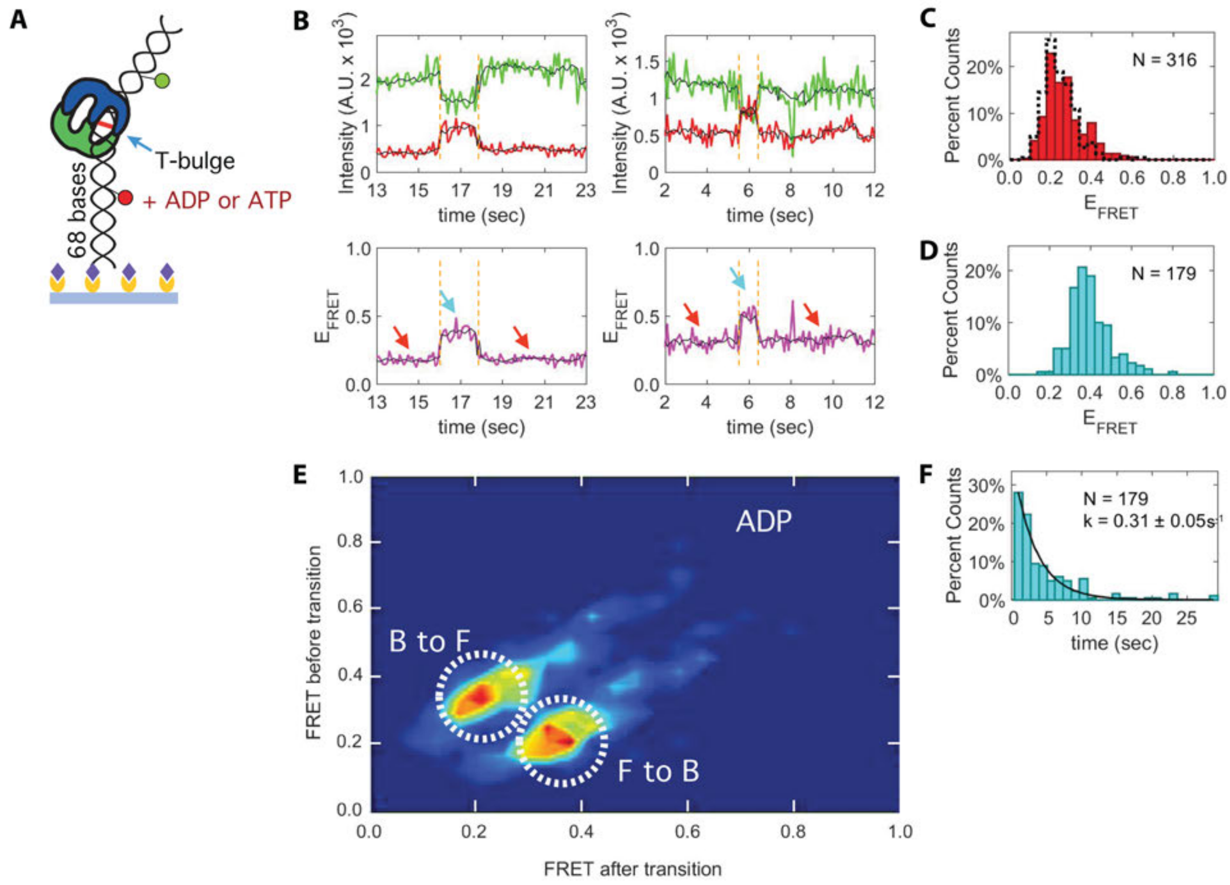

Figure S7: *Taq* MutS bends T-Bulge DNA to a single bent state in the presence of ADP. A) Schematic of a surface-attached 68-bp DNA molecule bound by *Taq* MutS at the single thymine insertion (T-bulge) (blue arrow). **(B)** Example donor (green) and acceptor (red) fluorescence time traces and the corresponding FRET efficiency (magenta) for experiments conducted at 10 nM *Taq* MutS and 2 mM ADP. The black line is the smoothed signal using a Chung-Kennedy filter. The red and cyan arrows denote low FRET and high FRET states, respectively. **C)** The distributions of FRET values for the low FRET states (red bars) and for DNA in the absence of *Taq* MutS (black dotted cityscape). **D)** The distribution of FRET values for the high FRET states (cyan bars). The high FRET distribution (centered on 0.36) represents a single bent DNA conformation induced upon MutS binding. **E)** Transition density plot (TDP) depicting the frequency of transitions between the low and high FRET states. The dominant transitions in the TDP are from low to high FRET (0.2 → 0.36, Free DNA (F) to Bent DNA (B)) and from high to low FRET (0.36 → 0.2, B to F). **F)** The distribution of dwell times for the high FRET state (cyan bars) fit to a single exponential function (τ = 3.2 s, black line).

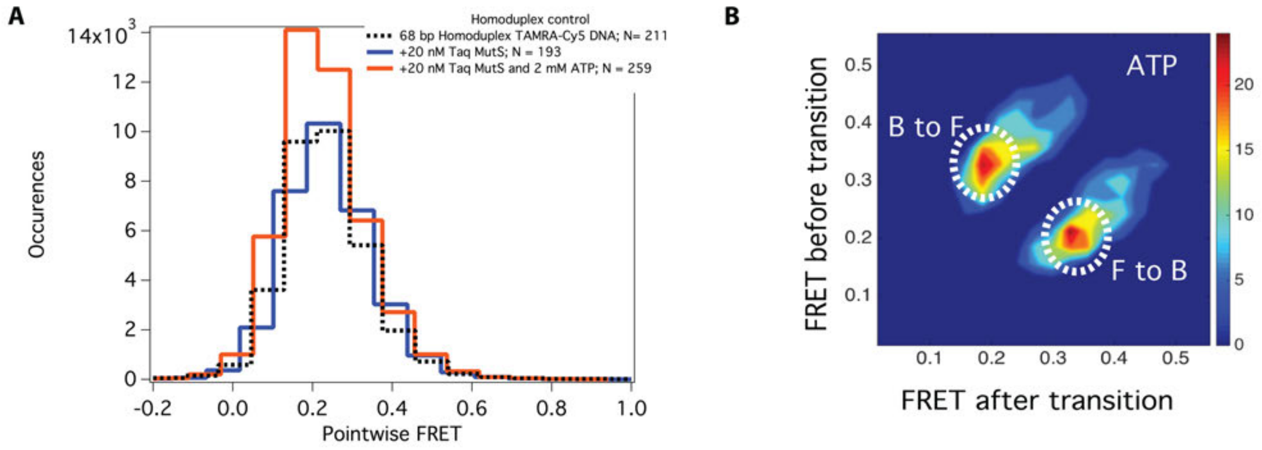

Figure S8: A) Homoduplex control for DNA-DNA FRET experiments (Figure 3-5 in the main text). We added 20 nM *Taq* MutS to surface-attached 68-bp homoduplex TAMRA-Cy5 DNA in the absence and presence of ATP. All three histograms are centered on FRET 0.2. In addition, no anti-correlated changes in donor and acceptor intensity are observed in individual traces, indicating that we cannot detect MutS bending the matched DNA. **B)** With the 68-bp T-bulge substrate and in the presence of ATP, ~70% of MutS binding events are ADP-like, bending DNA to a single state**.** In the TDP depicting the frequency of transitions between low and high FRET states, the dominant transitions are FRET 0.2 → 0.36 (F to B), and FRET 0.36 → 0.2 (B to F), as in the presence of ADP (Figure S7E). The FRET and dwell-time histograms of the single Bent DNA state in the presence of ATP appear in Figure 3F-G of the main text.

Supplementary Table 3: Parameters used to identify transitions in DNA bending traces using Gaussian Kernel and Chung-Kennedy algorithms (see Materials and Methods and Figure 4 in the main text).

User-defined Parameter	Value	Description
**Gaussian kernel (GK) edge detection**		A Gaussian derivative kernel of various widths is convolved with the FRET time traces
Threshold	0.4	Sets stringency of transition detection
Scales	8	Width of GK; dictates signal smoothing, precision of edge detection, and degree of noise suppression
**Chung-Kennedy (CK) edge detection**		Increases in the standard deviations for predictor windows are used to detect transitions
Windows (Predictor)	4	Number of data points forward and backward of a data point of interest for which a standard deviation is calculated
Exponent, *p*	10	The inverse of the standard deviation is raised to the exponent *p* to determine statistical weight of the average window
Percentile	98	Confidence level for local maxima identification

V.b. Statistics

Supplementary Table 4: Statistics for DNA-DNA FRET data presented in Figures 4 and 5. The extracted data from multiple experiment days was combined to generate the transition density plots and histograms. The table shows a breakdown of how many events from each experiment were included in the overall analysis.

**Protein Prep**	**Protein Conc.**	**2 states**	**3 states**	**4 states**	**5 states**	**Total multiple-state binding events in compiled data**	**% Multi-state binding events in compiled data**
2 (M88C)	20 nM, flow	8	12	1	0	21	14%
		38%	57%	5%	0%		
2 (M88C)	200 nM, flow	11	25	4	1	41	27%
		27%	61%	10%	2%		
2 (M88C)	200 nM, flow	5	14	3	0	22	14%
		23%	64%	14%	0%		
2 (M88C)	100 nM	3	18	2	1	24	16%
		13%	75%	8%	4%		
3 (E315C)	100 nM	8	32	5	0	45	29%
		18%	71%	11%	0%		
All		**2 states**	**3 states**	**4 states**	**5 states**	**TOTALS**	
		35	101	15	2	153	All multi-state
		23%	66%	10%	1%	100%	%
		**pre states**	**post states**			335	All single-state
		14	5			488	All events
		9%	3%			31%	% multi-state
						69%	% single-state
E315C		**2 states**	**3 states**	**4 states**	**5 states**	**TOTALS**	
		18%	71%	11%	0%	100%	
		**pre states**	**post states**			45	E315C multi-state
		6	0			116	E315C single-state
		13%	0%			161	E315C All events
						28%	% multi-state
						72%	% single-state

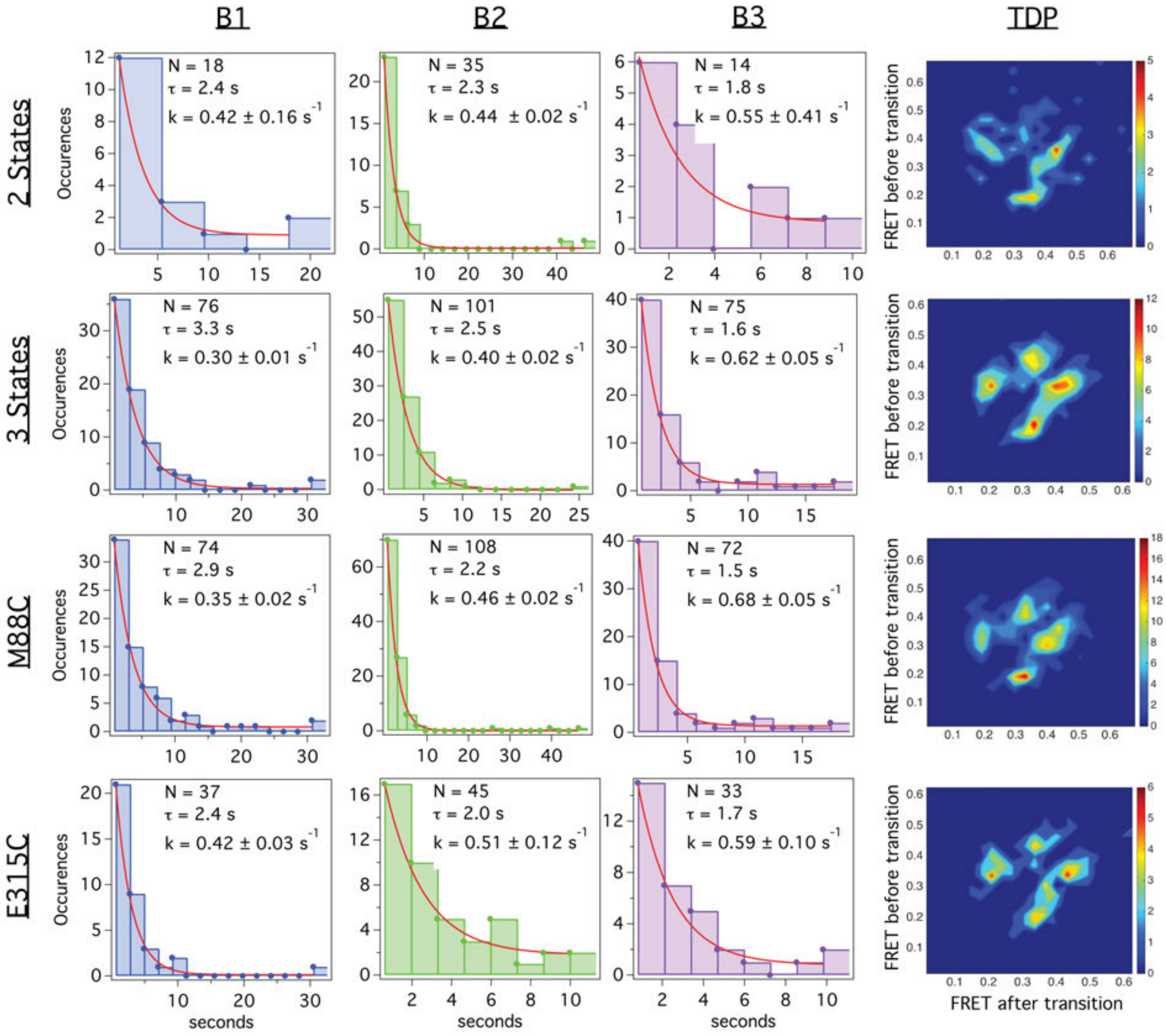

Figure S9: A kinetics breakdown for bent states 1-3. The compiled data are presented in Figures 4 and 5 in the main text. Row 1: binding events with 2 states; Row 2: binding events with 3 states; Row 3: binding events for experiments conducted with unlabeled M88C; Row 4: binding events for experiments conducted with unlabeled E315C. Each dwell-time histogram is fit to a single exponential (red lines), and the fit errors (± one standard deviation, IGOR Pro) for the rates are reported. A transition density plot for each category is also shown in column 4.

V.c. ATP

Independent replicate of DNA-DNA FRET experiments in the presence of ATP

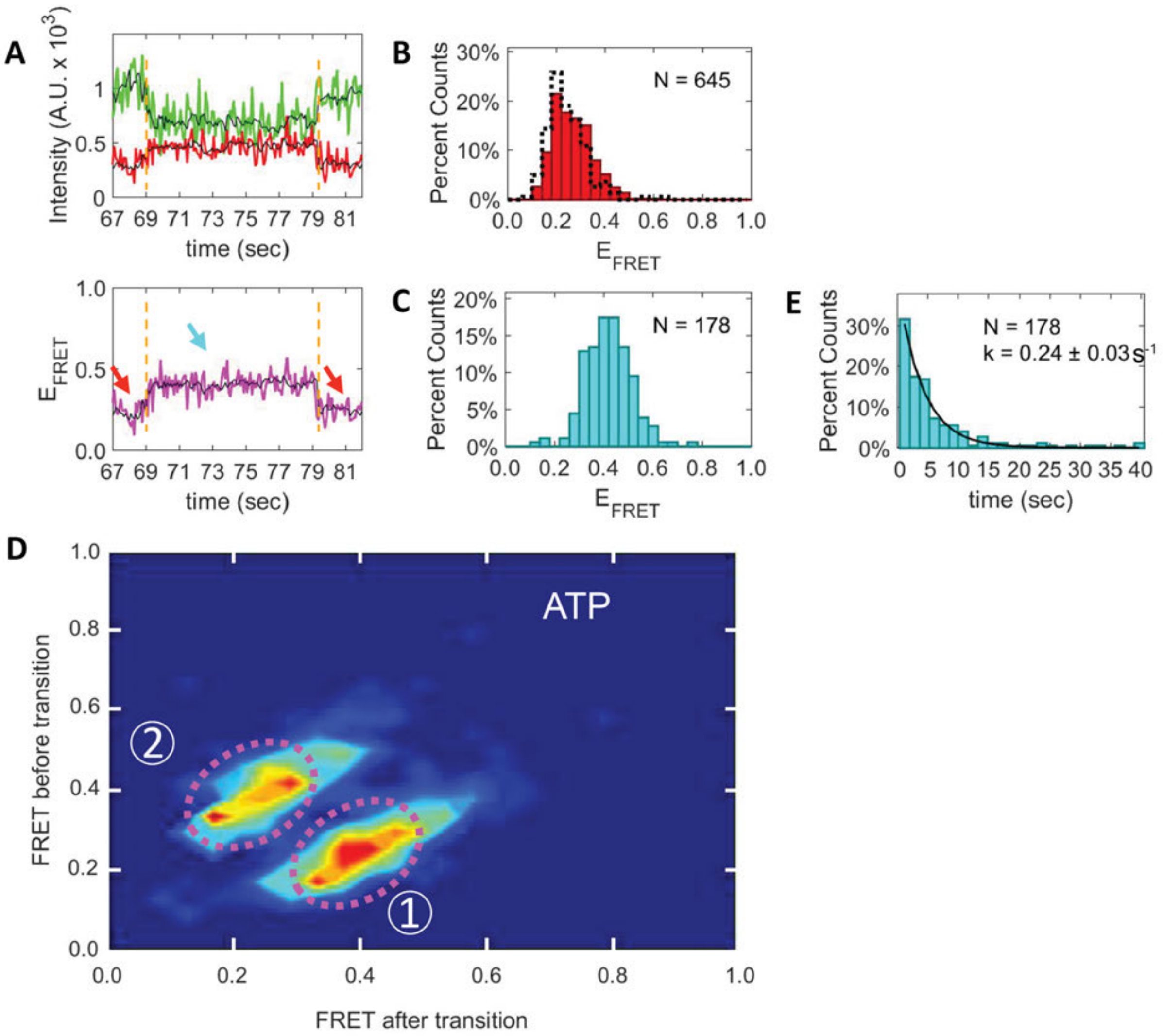

Figure S10: Independent replicate of DNA-DNA FRET experiments (Figures 4 and 5 in the main text). In the presence of ATP, 70% of binding events bend DNA to a single bent state (as with ADP). **A)** Example donor (green) and acceptor (red) fluorescence trace and its corresponding FRET efficiency (magenta) for experiments conducted at 10 nM *Taq* MutS and 2 mM ATP. The black line is the smoothed signal. Red and cyan arrows denote low FRET and high FRET states, respectively. **B)** The distributions of FRET values for the low FRET state (red bars) and for DNA in the absence of *Taq* MutS and nucleotide (black dotted cityscape). **C)** The distribution of FRET values for the high FRET state (cyan bars) centered on FRET 0.4. **D)** Transition density plot depicting the frequency of transitions between the low and high FRET states. **E)** The distribution of dwell times for the high FRET state (cyan bars) fit to a single exponential function (τ = 4.2 s, black line).

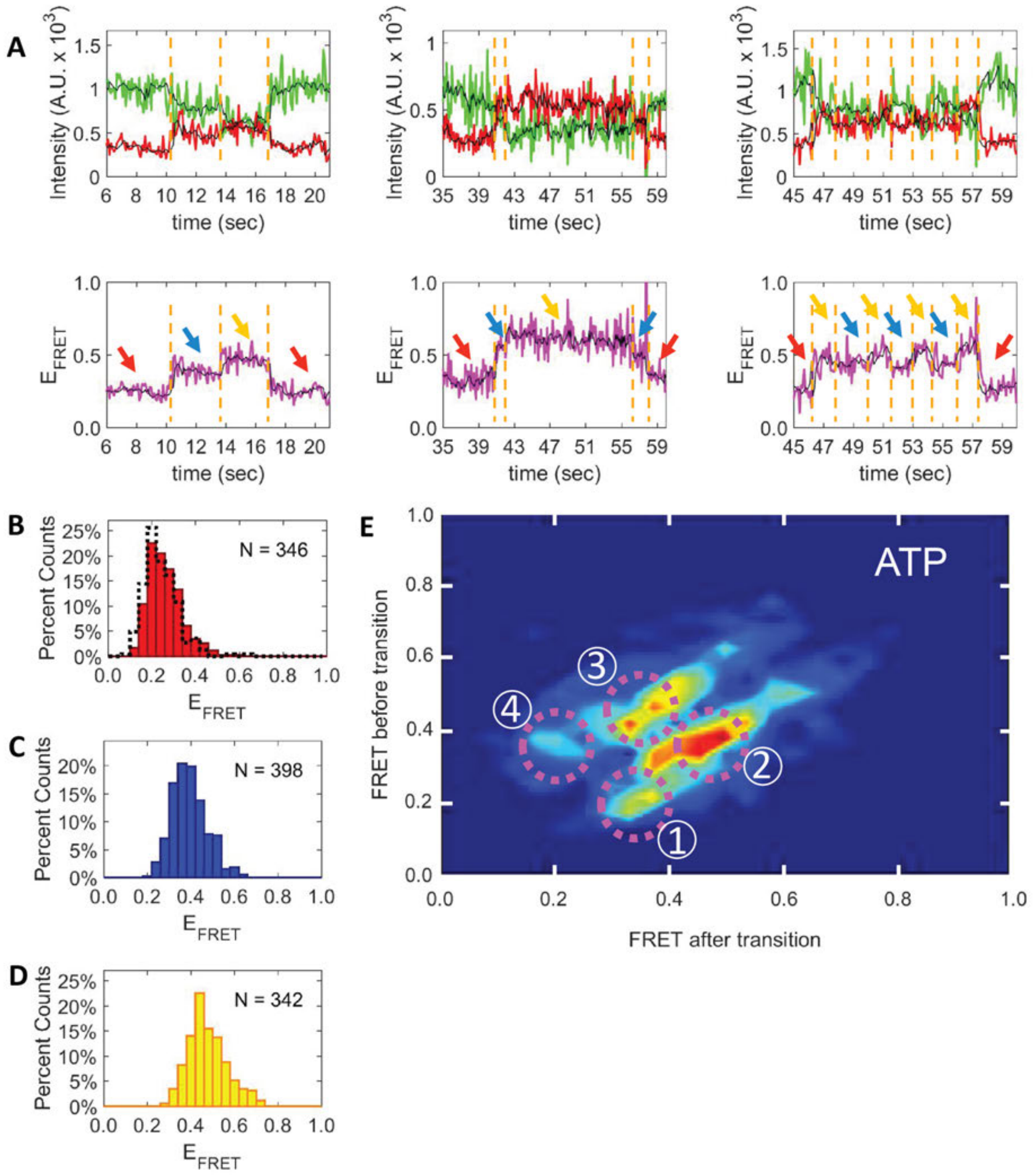

Figure S11: Independent replicate of DNA-DNA FRET experiments (Figures 4 and 5 in the main text). In the presence of ATP, 30% of binding events transition T-bulge DNA through a sequential pathway of conformations. A) Example donor (green) and acceptor (red) fluorescence traces and the corresponding FRET efficiency (magenta) for experiments conducted at 10 nM *Taq* MutS and 2 mM ATP. The black line is the smoothed signal. Red, blue and yellow arrows denote low, intermediate, and high FRET states, respectively, between the detected FRET transitions (dotted gold lines). **B)** The distributions of FRET values for the low FRET state (red bars) and for DNA in the absence of *Taq* MutS and nucleotide (black dotted cityscape). **C)** The distribution of FRET values for the intermediate FRET state (blue bars). **D)** The distribution of FRET values for the high FRET state (yellow bars). **E)** Transition density plot depicting the frequency of transitions between the low, intermediate and high FRET states (dotted magenta circles). (1) low FRET to intermediate FRET; (2) intermediate FRET to high FRET; (3) high FRET to intermediate FRET; and (4) intermediate FRET to low FRET.

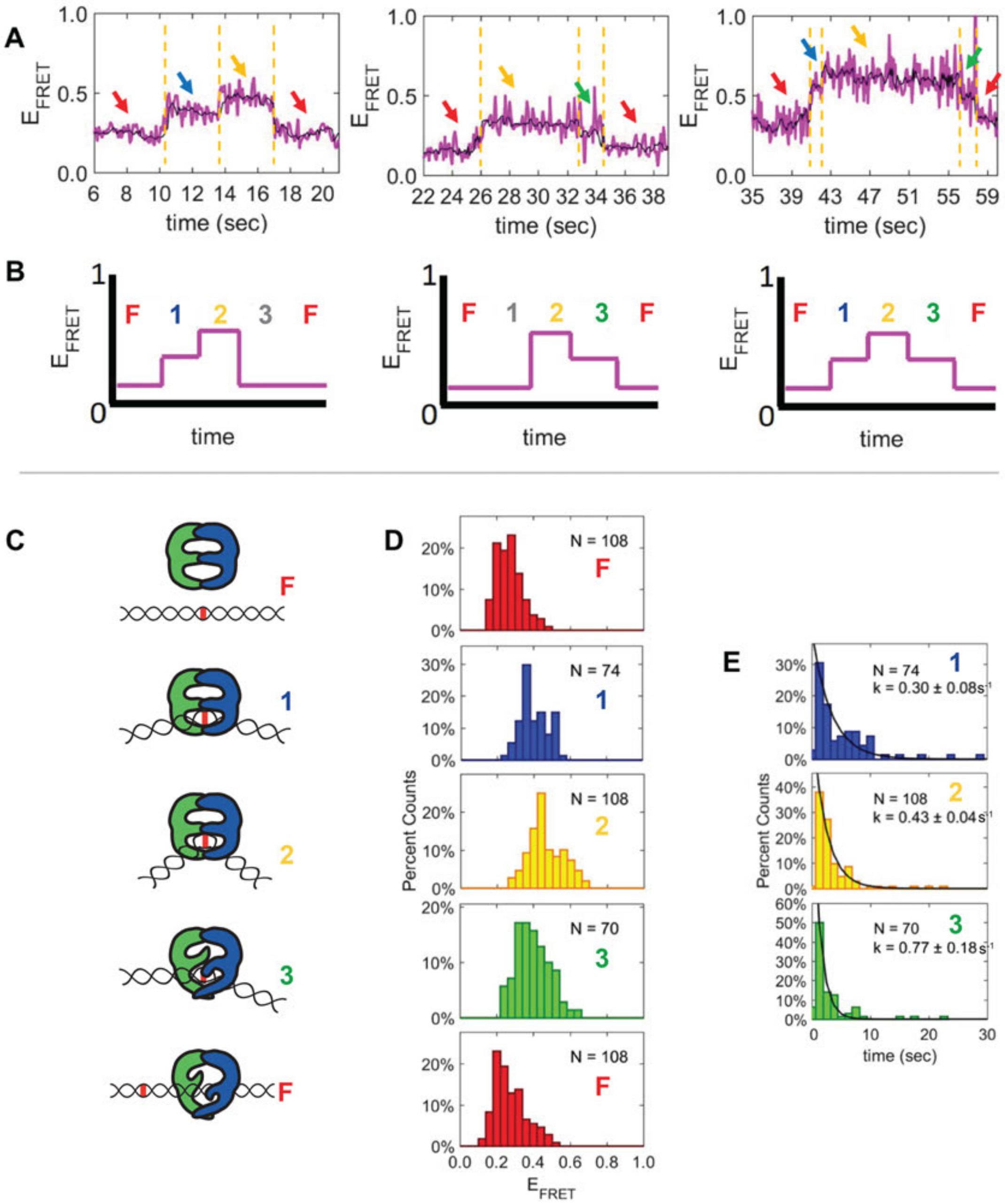

Figure S12: Independent replicate of DNA-DNA FRET experiments (Figures 4 and 5 in the main text). DNA bending in the presence of MutS and ATP follows a sequential pathway of bent states for 30% of binding events. A) Example FRET traces (magenta) for three multi-state binding events. The black line is the smoothed signal and the arrows point to FRET states between the detected FRET transitions (dotted gold lines). Throughout the figure, the F (Free), B1, B2, and B3 states are color-coded in red, blue, yellow, and green, respectively. **B)** Simplified schematics of the multi-state traces shown in A. **C)** Models depicting the DNA bending conformations through the F → B1 → B2 → B3 →F pathway. **D)** The distributions of FRET values for each state. **E)** The distributions of dwell times for each state fit to single exponential functions (τ (1) = 3.3 s, τ (2) = 2.3 s, τ (3) = 1.3 s, black lines).

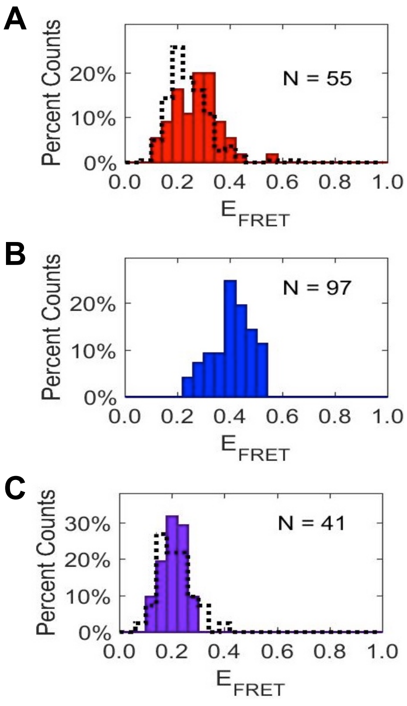

Figure S13: Control experiments in the absence of nucleotide and with homoduplex DNA.
A) The distribution of FRET values for the low FRET state from experiments conducted with T-bulge DNA and 10 nM *Taq* MutS in the absence of nucleotide (red bars), and the distribution of FRET values for T-bulge DNA in the absence of *Taq* MutS and nucleotide (black dotted cityscape). **B)** The distribution of FRET values for the high FRET state from experiments conducted with T-bulge DNA and 10 nM *Taq* MutS in the absence in nucleotide (blue bars). **C)** The distribution of FRET values from experiments conducted with homoduplex DNA in the presence (purple bars) and absence (dotted black cityscape) of 10 nM Taq MutS (purple bars).

Supplementary Table 5: Summary of DNA-DNA smFRET experiments with unlabeled MutS binding to TAMRA-Cy5 T-bulge DNA.

**FRET state**	**Replicate 1§**			**Replicate 2***			**Avg. ± Standard Deviation**		
	FRET	k (s^{-1})	τ (s)	FRET	k (s^{-1})	τ (s)	FRET	k (s^{-1})	τ (s)
Single bent (ATP)	0.4	0.24	4.2	0.35	0.31	3.2	**0.38** ± 0.04	**0.28** ± 0.05	**3.7** ± 0.7
B1	0.35	0.3	3.3	0.33	0.32	3.1	**0.34** ± 0.01	**0.31** ± 0.01	**3.2** ± 0.1
B2	0.45	0.43	2.3	0.41	0.44	2.3	**0.43** ± 0.03	**0.44** ± 0.01	**2.3** ± 0.0
B3	0.35	0.77	1.3	0.33	0.7	1.4	**0.34** ± 0.01	**0.74** ± 0.05	**1.4** ± 0.1

§First protein prep, combined data from experiments conducted on different days.
*Second and third protein preps, combined data from experiments conducted on 5 different days.

Supplementary Table 6: Kinetics for transitions reported in Figure 6 of the main text. Reported errors in normal black text are fit errors (± 95% confidence interval). Reported errors in blue italicized text are replicate errors. Fit errors for data from previous publications are the result of re-fitting. Biological replicates for individual experiments (protein-DNA and DNA-DNA) were performed with independent protein preps. Note that we are unable to observe the mobile clamp state in the 50 bp protein-DNA FRET experiments because the time to slide off of the free end of the DNA is near our imaging frame rate.

**Labeling**	**Bent State 1 (B1)**<br>τ<br>k	**Bent State 2 (B2)**<br>τ<br>k	**Bent State 3 (B3)**<br>τ<br>k	**Mobile clamp**<br>τ<br>k
**Intraprotein (M88C)** (ref. 3)	*2.5 s*<br>0.4 ± 0.4 s^{-1}	*2 s*<br>0.5 ± 0.5 s^{-1}	*1.3 s*<br>0.75 s^{-1}	*2.4 s*<br>0.4 s^{-1}
**Protein-DNA**				
M88C-50 bp (ref. 3)	4.2 s<br>0.24 ± 0.06 s^{-1}	0.8 s<br>1.3 ± 0.5 s^{-1}	1.2 s<br>0.8 s^{-1}	n/a
M88C-550 bp (ref. 3)	2 s<br>0.5 ± 0.4 s^{-1}	1.1 s<br>0.9 ± 1.0 s^{-1}	1.5 s<br>0.7 s^{-1}	2.2 s<br>0.45 s^{-1}
M88C-550 bp (ref. 4)	2 s<br>0.5 ± 0.1 s^{-1}	0.7 s<br>1.4 ± 0.6 s^{-1}	1.8 s<br>0.55 s^{-1}	2.2 s<br>0.45 s^{-1}
Avg. ± SD	*2.8 ± 1.2 s*	*0.9 ± 0.2 s*	*1.5 ± 0.3 s*	*2.2 ± 0.0 s*
E315C-550 bp, replicate 1	5.3 s<br>0.19 ± 0.80 s^{-1}	5.3 s<br>0.19 ± 0.80 s^{-1}	1.2 s<br>0.8 ± 0.2 s^{-1}	1.3 s<br>0.75 ± 0.15 s^{-1}
E315C-550 bp, replicate 2	5.6 s<br>0.18 ± 0.90 s^{-1}	5 s<br>0.2 ± 1.0 s^{-1}	1.2 s<br>0.83 ± 0.1 s^{-1}	1.5 s<br>0.65 ± 0.11 s^{-1}
Avg. ± SD	*5.5 ± 0.2 s*	*5.2 ± 0.2 s*	*1.2 ± 0.0 s*	*1.4 ± 0.2 s*
**DNA-DNA**				
replicate 1 (supplement)	3.3 s<br>0.30 ± 0.16 s^{-1}	2.3 s<br>0.43 ± 0.08 s^{-1}	1.3 s<br>0.77 ± 0.37 s^{-1}	n/a
replicate 2 (main text)	3.1 s<br>0.32 ± 0.03 s^{-1}	2.3 s<br>0.44 ± 0.01 s^{-1}	1.4 s<br>0.7 ± 0.2 s^{-1}	n/a
Avg. ± SD	*3.2 ± 0.1 s*	*2.3 ± 0.0 s*	*1.4 ± 0.1 s*	n/a

Supplementary references

1. Obmolova, G., Ban, C., Hsieh, P. and Yang, W. (2000) Crystal structures of mismatch repair protein MutS and its complex with a substrate DNA. *Nature*, **407**, 703-710.
2. Biswas, I. and Hsieh, P. (1996) Identification and characterization of a thermostable MutS homolog from Thermus aquaticus. *Journal of Biological Chemistry*, **271**, 5040-5048.
3. Qiu, R., DeRocco, V.C., Harris, C., Sharma, A., Hingorani, M.M., Erie, D.A. and Weninger, K.R. (2012) Large conformational changes in MutS during DNA scanning, mismatch recognition and repair signalling. *The EMBO journal*, **31**, 2528-2540.
4. Qiu, R., Sakato, M., Sacho, E.J., Wilkins, H., Zhang, X., Modrich, P., Hingorani, M., Erie, D.A. and Weninger, K.R. (2015) MutL traps MutS at a DNA mismatch. *Proceedings of the National Academy of Sciences*, **112**, 10914-10919.
5. Alani, E., Lee, J.Y., Schofield, M.J., Kijas, A.W., Hsieh, P. and Yang, W. (2003) Crystal structure and biochemical analysis of the MutS.ADP.beryllium fluoride complex suggests

a conserved mechanism for ATP interactions in mismatch repair. *J Biol Chem*, **278**, 16088-16094.

6. Jacobs-Palmer, E. and Hingorani, M.M. (2007) The effects of nucleotides on MutS-DNA binding kinetics clarify the role of MutS ATPase activity in mismatch repair. *Journal of Molecular Biology*, **366**, 1087-1098.
7. Sharma, A., Doucette, C., Biro, F.N. and Hingorani, M.M. (2013) Slow Conformational Changes in MutS and DNA Direct Ordered Transitions between Mismatch Search, Recognition and Signaling of DNA Repair. *Journal of Molecular Biology*, **425**, 4192-4205.
8. Antony, E. and Hingorani, M.M. (2004) Asymmetric ATP binding and hydrolysis activity of the Thermus aquaticus MutS dimer is key to modulation of its interactions with mismatched DNA. *Biochemistry*, **43**, 13115-13128.
